# Harnessing endogenous repair mechanisms for targeted gene knock-in of bovine embryos

**DOI:** 10.1038/s41598-020-72902-x

**Published:** 2020-09-29

**Authors:** Joseph R. Owen, Sadie L. Hennig, Bret R. McNabb, Jason C. Lin, Amy E. Young, James D. Murray, Pablo J. Ross, Alison L. Van Eenennaam

**Affiliations:** 1 Department of Animal Science, University of CA – Davis, Davis, CA USA; 2 Department of Population Health and Reproduction, School of Veterinary Medicine, University of CA – Davis, Davis, CA USA

**Keywords:** Biotechnology, Genetics

## Abstract

Introducing useful traits into livestock breeding programs through gene knock-ins has proven challenging. Typically, targeted insertions have been performed in cell lines, followed by somatic cell nuclear transfer cloning, which can be inefficient. An alternative is to introduce genome editing reagents and a homologous recombination (HR) donor template into embryos to trigger homology directed repair (HDR). However, the HR pathway is primarily restricted to actively dividing cells (S/G2-phase) and its efficiency for the introduction of large DNA sequences in zygotes is low. The homology-mediated end joining (HMEJ) approach has been shown to improve knock-in efficiency in non-dividing cells and to harness HDR after direct injection of embryos. The knock-in efficiency for a 1.8 kb gene was contrasted when combining microinjection of a gRNA/Cas9 ribonucleoprotein complex with a traditional HR donor template or an HMEJ template in bovine zygotes. The HMEJ template resulted in a significantly higher rate of gene knock-in as compared to the HR template (37.0% and 13.8%; *P* < 0.05). Additionally, more than a third of the knock-in embryos (36.9%) were non-mosaic. This approach will facilitate the one-step introduction of gene constructs at a specific location of the bovine genome and contribute to the next generation of elite cattle.

## Introduction

The use of genetics, selective breeding and reproductive technologies has had a positive impact on livestock genetic improvement^1^. Introducing useful variants using traditional crossbreeding approaches is slow, and is associated with unwanted linkage drag^2^. Genome editing offers an opportunity to augment livestock breeding programs by directly introducing targeted genetic variation to improve desired traits such as disease resistance and resilience^2–4^. This would help to translate the extensive investment in livestock genome sequencing and variant discovery into applied genetic improvement outcomes^4^.

In livestock, the predominant method for creating a gene insertion or knock-in (KI) using genome editing uses a two-step process, in which first a primary cell line is modified in culture and then used for somatic cell nuclear transfer (SCNT) cloning to create an animal with the intended gene modification^5^. However, SCNT cloning is generally inefficient, and production of viable calves can be highly variable^6^. An alternative approach for increasing the efficiency of producing genome edited livestock is through direct cytoplasmic injection (CPI) of in vitro fertilized embryos^7^. However, the use of standard embryo culture and gene KI techniques by using CPI often results in low integration rate, and/or embryos displaying high levels of genetic mosaicism^8^.

In cattle, there have been no successful KIs of large segments of DNA using CPI of one-cell embryos and only two reports showing the successful introduction of single nucleotide polymorphisms (SNPs) or precise deletions in bovine embryos^9,10^. In addition, high rates of mosaicism are often observed due to the time at which editing reagents are introduced, as we have reported when introducing CRISPR/Cas9 editing reagents into bovine embryos at 18 h post insemination (hpi), particularly when using Cas9 mRNA^11^. However, a recent study showed that introducing editing reagents into MII oocytes or early bovine zygotes (10 hpi), prior to the first S-phase, reduced mosaicism^12^.

Many attempts have been made to increase the rate of homologous recombination (HR), or decrease the rate of non-homologous end joining (NHEJ) for gene insertion, when using the CRISPR/Cas9 system via CPI of zygotes^13^. However, these approaches have been unsuccessful in bovine embryos as HR is primarily restricted to actively dividing cells^14^. In the early embryo, the primary repair mechanism for DNA double-strand breaks (DSBs) is the NHEJ repair pathway, which is a result of the low concentration of proteins necessary for HR and leads to repair by blunt-end ligation^14^. Recently, an alternative homology directed repair (HDR) approach was utilized for KI using a donor template via the homology-mediated end joining (HMEJ) method, which contains the KI gene of interest flanked by 800–1000 bp homology arms and the CRISPR target site outside each homology arm^15^. This method was shown to be successful in gametes and early one-cell embryos, in which proteins necessary for pushing DNA repair machinery towards the end-joining pathways are at their highest concentration^13^.

While the mechanisms responsible for HDR-mediated repair of donor templates using HMEJ have yet to be fully elucidated, this approach is thought to utilize multiple repair pathways^15^. Given the 800–1000 bp homology arms used in the design of the HMEJ donor template, the possibility of HDR integration of the gene of interest by proteins involved in the HR repair pathway exists. Another possibility is DSB repair by the microhomology-mediated end joining (MMEJ) pathway. This pathway uses the alignment of microhomologous (5–18 bp) regions of DNA to repair DSB, using proteins similar to those involved in the NHEJ pathway^16^. The addition of the CRISPR target sites in the HMEJ donor template results in the donor template being cut alongside the target site in the genome. It has been hypothesized that this leaves homologous regions open to repair using the MMEJ pathway utilizing 800–1000 bp regions of homology rather than the 5–18 bp typically used^15^. In addition, while HR is restricted to the actively dividing cell, MMEJ is thought to be active throughout the cell cycle^16^. Finally, the donor template being cut alongside the target site within the genome, there is a possibility for DSB repair by blunt-end ligation, similar to the homology independent targeted insertion (HITI) approach^17^. The HITI method uses a donor template containing a gene of interest flanked by the CRISPR target sites without any homology arms. The target sites within the donor template are cleaved alongside the genomic target site and the gene of interest is inserted by blunt-end ligation via NHEJ repair mechanisms. While the HMEJ approach does uses homology arms, placement of the CRISPR target sites outside the homology arms could result in cleaving of the donor template and insertion of the gene of interest, along with the homology arms, into the cut site by blunt-end ligation, resulting in homology-independent insertion (HII).

In this study, we employed the HMEJ method for the targeted insertion of the sex-determining region Y (*SRY*) gene into a region 10 kb downstream of the zinc finger, X-linked (*ZFX*) gene on the X chromosome of bovine embryos by microinjecting CRISPR/Cas9 editing reagents into either in vitro matured oocytes prior to in vitro fertilization, or presumptive zygotes 6 hpi. We used two donor templates to compare KI efficiency using the HMEJ and HR approaches, and show increased rate of gene insertion at the target location and a decreased rate of mosaicism with the HMEJ approach.

## Results

### Guide-RNA design, testing and selection

Each of three guide-RNAs (gRNAs) targeting the ZFX locus were independently injected 6 hpi alongside Cas9 protein into zygotes in groups of 30, including a non-injected group of 50 embryos as controls. Each treatment was repeated three times. The overall number of blastocysts and mutation rate was determined for each guide injected into zygotes (Supplemental Table S1). ZFXg3 showed a significantly higher mutation rate (81.8%) compared to ZFXg1 and ZFXg2 (37.5% and 57.1%, respectively; *P* < 0.05). In addition, a significant decrease in development to the blastocyst stage was observed when comparing ZFXg2 and ZFXg3 injected embryos to control non-injected embryos (17.5% and 14.9 vs. 26.7%, respectively; *P* < 0.05). However, there was no significant difference between the development to the blastocyst stage when comparing ZFXg1 injected embryos to non-injected controls (25.8% vs. 26.7%; *P* > 0.05). Based on these data, ZFXg3 was selected for further embryo KI testing.

### Donor plasmid testing in bovine oocytes and zygotes

**Figure 1:**
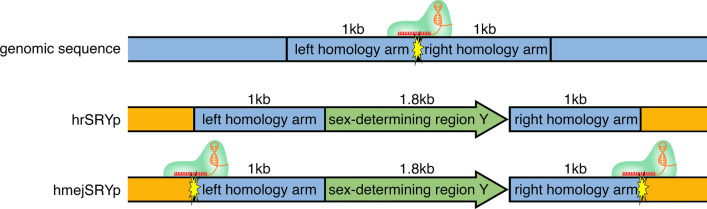
Schematic representation of donor plasmids used to test knock-in efficiency in bovine embryos. hrSRYp was used to compare the homologous recombination (HR) approach. hmejSRYp was used compare the homology mediated end-joining (HMEJ) approach. *SRY* = sex determining region Y; Yellow starburst = gRNA target site with gRNA/Cas9 ribonucleoprotein complex bound.

**Figure 2:**
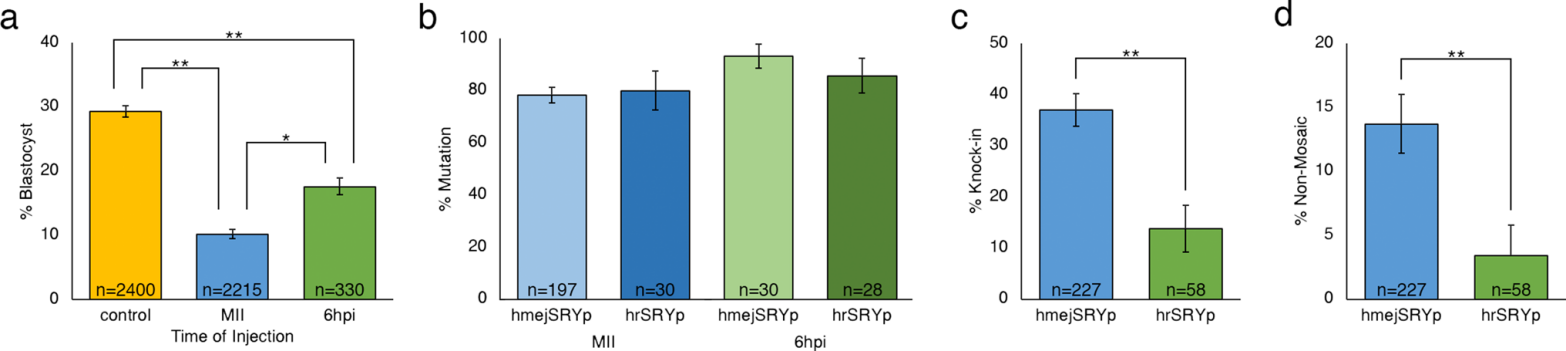
Comparison of development rates and knock-in efficiencies when microinjecting gRNA/Cas9 ribonucleoprotein editing reagents and hmejSRYp or hrSRYp templates into mature oocytes or presumptive zygotes six hours after insemination (6 hpi). (**a**) Percentage of all embryos that reached the blastocyst stage for oocytes injected prior to fertilization (MII) or presumptive zygotes injected 6 hpi as compared to uninjected control. (**b**) Breakdown by donor template and time of injection of mutation rate in blastocysts (**c**) Breakdown by donor template of knock-in rate in blastocysts. (**d**) Percent non-mosaic knock-in blastocysts when comparing donor template. Error bars = standard error of the mean. *P < 0.05; **P < 0.005.

The HMEJ (hmejSRYp) and HR (hrSRYp) donor plasmids (Fig. 1) were each injected alongside ZFXg3 and Cas9 protein to determine KI efficiency in bovine embryos. The editing reagents were injected into 25 groups of 30 MII oocytes each 18 h after maturation followed by in vitro fertilization, and into eight groups of 30 presumptive zygotes each 6 hpi. A significant decrease in development to the blastocyst stage was again observed when injecting the *SRY* donor plasmids in MII oocytes and zygotes 6 hpi as compared to non-injected controls (10.2% and 17.6% vs. 29.3%, respectively; *P* < 0.01; Fig. 2a). In addition, there was a significant decrease in development to the blastocyst stage when injecting MII oocytes as compared to presumptive zygotes 6 hpi (10.2% vs. 17.6%; *P* < 0.05).

Each blastocyst from the injected groups was analyzed for mutations induced at the target site, presence of KI and sex (Supplementary Information, Fig. S1). Mutation rates were not significantly different between male and female embryos, donor plasmids, or time of injection (*P* > 0.05; Table 1; Fig. 2b).

**Table 1:** Mutation, knock-in, and mosaicism rate in blastocysts after cytoplasmic injection of the gRNA:Cas9 RNP complex and hmejSRYp or hrSRYp in the MII oocyte or presumptive zygotes 6 h post insemination.

Sex	TOI	Donor	Total embryos	% Blastocysts (n)	% Mutation (n)	% Total KI (n)	% Non-mosaic KI (n)	Subset of knocked-in embryos		
								Non-mosaic		% Mosaic (n)
								% Homo/ Hemi (n)	% HII (n)	
Female	MII	HMEJ	960	8.5 (82)	82.9 (68)	41.5 (34)^a^	9.8 (8)^a^	17.6 (6)^a^	5.9 (2)^a^	76.5 (26)^a^
		HR	125	11.2 (14)	78.6 (11)	28.6 (4)^b^	14.3 (2)^a^	50.0 (2)^a^	0.0 (0)^a^	50.0 (2)^a^
	6 hpi	HMEJ	85	15.3 (13)	92.3 (12)	46.2 (6)^a^	15.4 (2)^a^	16.7 (1)^a^	16.7 (1)^a^	66.7 (4)^a^
		HR	95	16.8 (16)	87.5(14)	18.8 (3)^b^	0.0 (0)^a^	0.0 (0)^a^	0.0 (0)^a^	100.0 (3)^a^
Male	MII	HMEJ	1005	11.4 (115)	74.8 (86)	33.0 (38)^a^	14.8 (17)^a^	21.1 (8)^a^	23.7 (9)^b^	55.3 (21)^b^
		HR	125	12.8 (16)	81.3 (13)	6.3 (1)^b^	0.0 (0)^a^	0.0 (0)^a^	0.0 (0)^a^	100.0 (1)^b^
	6 hpi	HMEJ	85	20.0 (17)	94.1 (16)	35.3 (6)^a^	23.5 (4)^a^	16.7 (1)^a^	50.0 (3)^b^	33.3 (2)^b^
		HR	65	18.5 (12)	83.3 (10)	0.0 (0)^b^	n/a	n/a	n/a	n/a
Total	MII	HMEJ	1965	10.0 (197)	78.2 (154)	36.5 (72)^a^	12.7 (25)^b^	19.4 (14)	15.3 (11)	65.3 (47)
		HR	250	12.0 (30)	80.0 (24)	16.7 (5)^b^	6.7 (2)^a^	40.0 (2)	0.0 (0)	60.0 (3)
	6 hpi	HMEJ	170	17.6 (30)	93.3 (28)	40.0 (12)^a^	20.0 (6)^b^	16.7 (2)	33.3 (4)	50.0 (6)
		HR	160	17.5 (28)	85.7 (24)	10.7 (3)^b^	0.0 (0)^a^	0.0 (0)	0.0 (0)	100.0 (3)
Control	Not injected		2400	29.3 (702)	–	–	–	–	–	–

Letters that differ in the same column are significantly different (*P* < 0.05).

*TOI* time of injection, *HMEJ* hmejSRYp, *HR* hrSRYp, *KI* knock-in, *Homo* homozygous, *Hemi* hemizygous, *HII* homology independent insertion.

**Figure 3:**
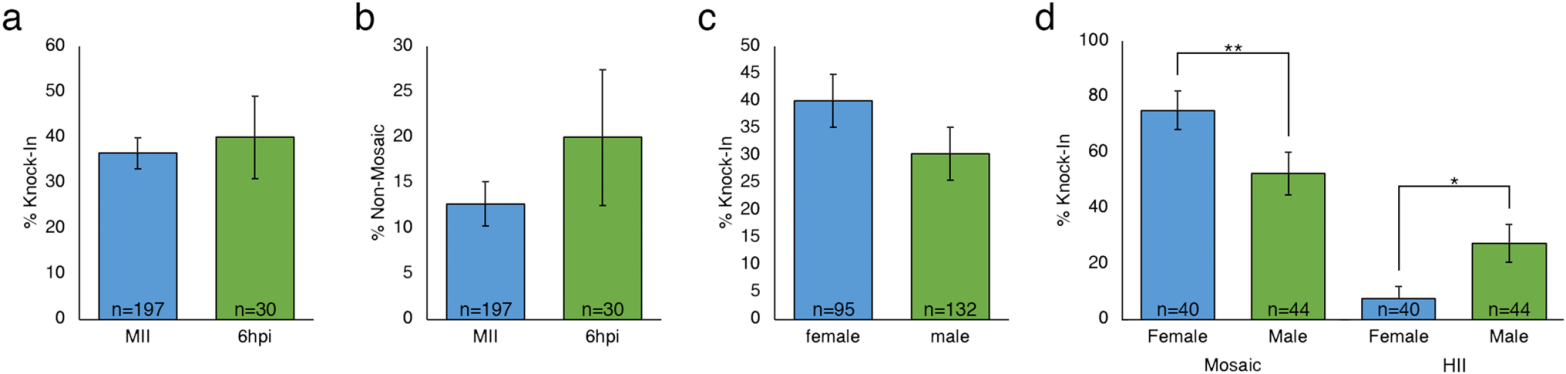
Comparison of development rates and knock-in efficiencies when microinjecting gRNA/Cas9 ribonucleoprotein editing reagents and hmejSRYp template into mature oocytes or presumptive zygotes six hours after insemination (6hpi) (**a**) Percent knock-in blastocysts when comparing time of injection. (**b**) Percent non-mosaic knock-in blastocysts when comparing time of injection. (**c**) Percent knock-in blastocysts when comparing sex of the embryo. (**d**) Evaluation of knock-in blastocysts by type of knock-in based on sex of the embryo. HII = homology independent insertion. Error bars = standard error of the mean. *P < 0.05; **P < 0.005.

The KI efficiency was significantly increased (*P* < 0.01) for the hmejSRYp donor plasmid (37.0%) compared to the hrSRYp donor plasmid (13.8%) (Fig. 2c), with 36.9% of the KIs being non-mosaic. The proportion of embryos that resulted in a non-mosaic KI blastocyst was significantly higher for hmejSRYp compared to hrSRYp (13.7% versus 3.4%, respectively; *P* < 0.01; Fig. 2d). However, there was no significant difference in KI efficiency when comparing blastocysts that were injected at the MII stage to presumptive zygotes injected 6 hpi (36.5% and 40.0%, respectively; *P* > 0.05; Fig. 3a). In addition, there was no significant difference in the number of non-mosaic embryos when injecting the hmejSRYp donor plasmid at the MII stage compared to 6 hpi (12.7% vs. 20.0%, respectively; Fig. 3b; p > 0.05).

Along with the presence of homozygous and hemizygous KI embryos resulting from HDR, there were 15 homozygous or hemizygous embryos that resulted from HII: 11 in MII injected oocytes (15.3%) and four in embryos injected 6 hpi (33.3%; Table 1). This type of insertion is characterized by the presence of repeating homology arms at the target site. Embryos were analyzed for the presence of the donor plasmid backbone sequence that could likewise have been integrated by HII, but no sequencing reads containing backbone sequences were found (Supplementary Information, Fig. S2). In addition, there was no alignment of the unsorted reads to the donor plasmid backbone.

There was no significant difference in KI efficiency based on sex of the embryo (40.0% female vs. 30.3% male; *P* > 0.05; Fig. 3c). However, there was a significant increase in the level of female mosaic KI embryos compared to male mosaic KI embryos (75.0% vs. 52.3%; *P* < 0.01; Fig. 3d). Additionally, there was an increased rate of blunt-end ligation of the cleaved hmejSRYp donor plasmid by HII KI in male embryos compared to female embryos (27.3% vs. 7.5%; *P* < 0.05; Fig. 3d).

### Evaluation of mosaicism

**Figure 4:**
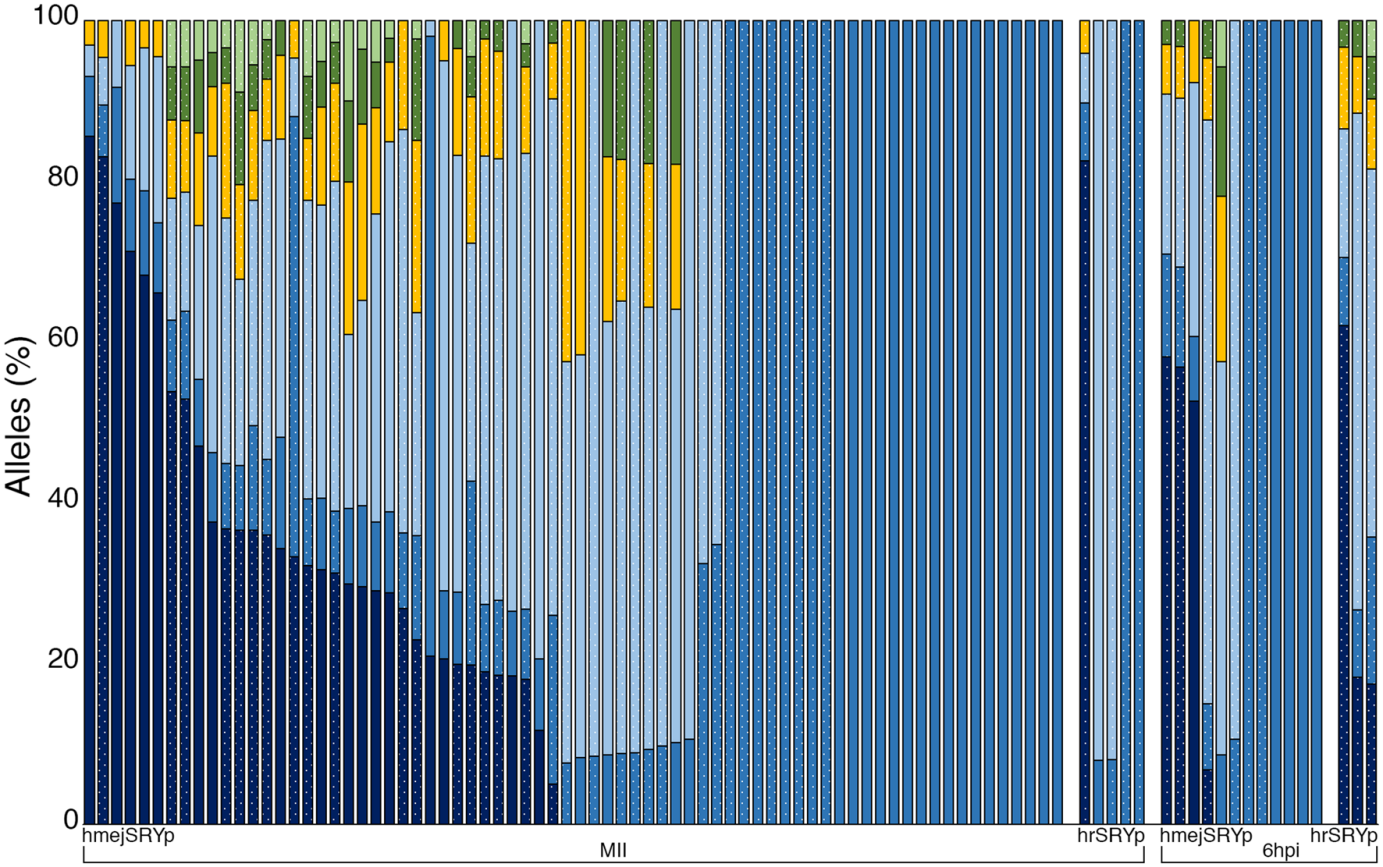
Bar graph depicting the percentage of alleles determined by PacBio sequencing in each of the 92 blastocysts that contained the SRY knock-in band in PCR analysis. Microinjection occurred either prior to fertilization (MII oocytes) or into presumptive zygotes 6 h post insemination with either hmejSRYp or hrSRYp donor templates. Samples contained some combination of the wild type allele (dark blue), SRY knock-in (blue) or an allele containing an insertion or deletion mediated by non-homologous end joining (light blue, yellow, dark green and light green). Dotted bars are female; solid bars are male.

Blastocysts that contained the *SRY* KI based on PCR analysis (Supplementary Information, Fig. S1 lanes 1–4) from each of the four injected groups were evaluated for the level of mosaicism using PacBio sequencing (Fig. 4; Supplementary Information, Table S2). Overall, there was a 64.1% rate of mosaicism. There was no significant difference in the average number of alleles, proportion of wild type reads or proportion of *SRY* KI reads between MII oocytes and presumptive zygotes injected with the hmejSRYp donor plasmid, and MII oocytes injected with the hrSRYp donor plasmid (Table 2). However, there was a significant increase in the number of alleles and the proportion of wild type reads and a significant decrease in *SRY* KI reads in the three 6 hpi presumptive zygotes injected with the hrSRYp donor plasmid, compared to all other groups.

**Table 2:** Average number of alleles, percent of wild type reads and percent of *SRY* knock-in reads by time of injection (MII oocytes or presumptive zygotes 6 h post insemination) and donor template (hmejSRYp or hrSRYp).

TOI	Donor	Alleles	SEM	% Wild type	SEM	% SRY	SEM
MII	HMEJ	3.3^a^	± 0.240	17.9^a^	± 2.8	43.4^a^	± 5.0
	HR	2.0^a^	± 0.546	16.5^a^	± 16.5	44.7^a^	± 22.6
6 hpi	HMEJ	2.7^a^	± 0.554	14.5^a^	± 7.2	55.1^a^	± 13.6
	HR	5.3^b^	± 0.335	32.6^b^	± 14.7	11.7^b^	± 3.3

Letters that differ in the same column are significantly different (*P* < 0.05).

*TOI* time of injection, *HMEJ* hmejSRYp, *HR* hrSRYp, *SEM* standard error of the mean.

Notably, within the group of 72 blastocysts derived from injected MII oocytes there were 35% (8 female and 17 male) non-mosaic KIs with the hmejSRYp donor plasmid (Table 1). Of these, two and nine were HII, respectively. One of the female embryos contained roughly 50% *SRY* KI reads, suggesting a monoallelic KI. In addition, the wild type allele was also present, as well as two additional alleles containing indels for that sample, resulting in a mosaic blastocyst. Within this same group, two female embryos contained ~ 35% *SRY* KI reads with the remaining reads containing a ten base pair deletion at the CRISPR/Cas9 cut site. One of the male embryos contained ~ 75% *SRY* KI reads, suggesting that the insertion in one chromosome occurred after DNA replication in the zygote or in the 2-cell embryo, and that the other occurred after DNA replication at the 2-cell stage or at the 4-cell stage. In addition, two of the male embryos within this group contained ~ 25% *SRY* KI reads indicating that there was likely an insertion of *SRY* into one of the chromosomes after DNA replication at the 2-cell stage or at the 4-cell stage prior to DNA replication. The remaining mosaic samples across all four groups contained less than 20% *SRY* KI reads with each of them containing more than three alleles.

### Embryo transfers

Following analysis of blastocysts identifying the successful KI of *SRY* into the X chromosome, recipient animals were synchronized for embryo transfer of HMEJ donor injected MII oocytes followed by in vitro fertilization. For the first trial, a total of ten day-eight blastocysts were transferred to ten synchronized heifers (Supplementary Table S3). Embryo biopsies were taken prior to transfer and analyzed after embryo transfer. Three of the ten biopsies were positive for *SRY* insertion into the X chromosome. Two of the three were potential heterozygous/mosaic females and the third was a mosaic male. At day 35 of embryo development, pregnancies were diagnosed by transrectal ultrasonography, showing none of the recipients were positive for pregnancy. To limit the transfer of blastocysts that did not contain the *SRY* insertion, *SRY* KI blastocysts were produced over several rounds of MII oocyte injection, biopsied at the blastocyst stage and vitrified. A total of 12 *SRY* positive blastocysts were then used for embryo transfer. In addition, six non-biopsied fresh day-eight blastocysts produced from MII oocytes injected with the HMEJ donor were transferred on the same day. Three of the twleve recipients that received vitrified blastocysts and two of the six that received fresh day eight blastocysts were tentatively pregnant at day 35 based on ultrasound but were subsequently determined to be not pregnant at day 42.

## Discussion

Using the HMEJ approach^15^ allowed for the targeted insertion of a large gene construct into the X-chromosome of bovine embryos. In 2017, Yao et al.^15^, showed that the HMEJ approach resulted in an increase in KI efficiency in mouse embryos to 22.7% compared to 11.9% when utilizing the MMEJ approach, 3.3% when utilizing HR, and 1.4% when using HITI. Using a HMEJ donor template, we observed a similar increase in KI efficiency (37.0%) compared to methods utilizing the traditional HR template (13.8%; ~ 2.7X increase). Our HR template KI results are similar to efficiencies previously reported in livestock species for zygote cytoplasmic injection of an HR template (5.7% in sheep embryos^18^ and 6.5% in pig embryos^19^). While there have been reports of using genome editors and donor template ODNs to induce SNPs or precise deletions of bovine embryos^9,10^, this is the first reported use of the CRISPR/Cas9 system for efficient KI of a large DNA segment (1.8 kb).

To determine the optimal time for delivery of editing reagents to obtain non-mosaic KI embryos, we compared injection of the mature MII oocytes prior to fertilization to injection of zygotes 6 hpi. In bovine embryos, DNA replication starts approximately 13 hpi. In our system, we achieved acceptable rates of development with only 6 h of gamete coincubation. The advantage of introducing editing reagents into the early zygote is the likelihood of reducing mosaicism by editing prior to the onset of DNA synthesis. Given that, we chose to introduce the editing reagents as early as possible (MII oocytes) and as late as feasible (6 hpi) while still attempting to limit mosaicism.

Injecting at these time points significantly reduced the number of embryos that reached the blastocyst stage compared to control non-injected embryos (10.2% and 17.6% compared to 29.3%). Microinjection itself has not been found to have a significant effect on bovine embryonic development^20^, but we observed a decrease in the number of embryos that reached the blastocyst stage as the rate of mutation for a given guide increased (Supplemental Table, S1). We previously observed a similar inverse correlation between the number of embryos that reached the blastocyst stage and guide mutation efficiency^11^. In addition, there was a further decrease in the percentage of embryos that reached the blastocyst stage when comparing injected MII oocytes (10.2%) to zygotes injected 6 hpi (17.6%) likely due to the decreased fertilization rate of cumulus free oocytes, even when co-incubated with cumulus-oocyte-complexes (COCs).

There was little difference observed in KI efficiency between injected MII oocytes and zygotes injected 6 hpi (36.5% and 40.0%, respectively; *P* > 0.05). While the sample size of zygotes injected 6 hpi is low; there were proportionally fewer embryos that reached the blastocyst stage when injecting MII oocytes compared to 6 hpi. Likewise, a previous study found efficiencies after introducing editing reagents into MII oocytes or 10 hpi zygotes^12^. Collectively, these results suggest it may be most beneficial to inject zygotes 6 hpi as the KI efficiency is comparable to injecting MII oocytes, but the former yields roughly five percent more blastocysts per group.

The overall KI efficiency was higher using the HMEJ approach compared to the HR approach. Additionally, the rate of obtaining a non-mosaic embryo was increased using the HMEJ approach. This increased rate of non-mosaic integration of *SRY* into the X chromosome (13.7%) is likely due to the DSB potentially being repaired by multiple repair mechanisms. This approach can potentially recruit HDR and MMEJ-mediated repair pathways, as well as HII KIs. For the goal of inserting the 1.8 kb *SRY* gene with promoter and coding sequence, the orientation of the insert is inconsequential, as transcription is not strand specific. However, in cases where precise changes, such as allelic substitutions or tagging a protein, are the ultimate goal, orientation of the insert is critical.

While HDR approaches have achieved successful KIs in cell lines, little success has been reported in livestock embryos. This is primarily due to the inactivity of the HR pathway in gametes and one-cell embryos prior to the first round of DNA replication^13^ and the low efficiency of integration using the HITI approach^15^. The HMEJ approach utilized in this study has the ability to use the HDR pathways, the microhomology-mediated end joining (MMEJ) pathway and the NHEJ pathway, resulting in a HII KI. The later being observed here with 15 homozygous/hemizygous HII KI embryos (6.6%)^15^. While the significant increase in mosaicism in female embryos can likely be attributed to the presence of two X chromosomes compared to only one in the male embryos, the significant increase in HII KI male embryos was unexpected.

Approaches that increased large KI efficiency in mouse embryos, including pronuclear microinjection of zygotes during S-phase prior to activation of the HR pathway^21^ and injection at the two-cell stage^22^, are unlikely to be directly translatable to bovine embryos. Technically, in contrast to mice and rats, bovine zygotes have a very dark cytoplasm, limiting visualization of the pronucleus. Furthermore, the increased KI rate at the two-cell stage in mice is likely due to the long G2 phase as a result of genome activation. This approach is unlikely to achieve similar results in bovine embryos given that genome activation does not occur until the eight- to 16-cell stage in this species^23^. Moreover, although both of these approaches have shown an increased rate of KI efficiency in mouse embryos, neither study evaluated the level of mosaicism in embryos or offspring, which is likely to be high given that both methods target embryos after the first round of DNA replication has started or been completed. The production of non-mosaic germline alterations is important in uniparous species like cattle as their two-year generation interval makes breeding mosaic founders to produce heterozygous or homozygous animals expensive and time consuming.

The efficient integration of *SRY* into bovine embryos using the CRISPR/Cas9 approach has shown an overall improvement compared to previously reported studies. In a previous study, introducing CRISPR/Cas9 editing reagents into bovine embryos 18 hpi, resulted in an average level of mosaicism of 94.2%^11^. Early delivery of CRISPR/Cas9 reagents combined with immediate and short activity of Cas9 reduced mosaicism^24^. Here we found a decreased level of mosaicism after introducing CRISPR/Cas9 editing reagents into in MII oocytes (64.9%) and presumptive zygotes 6 hpi (60.0%) (Table 2). These results are not as low as the 30% reported when introducing targeted gene knock-outs in 6 hpi zygotes^12^. However, in this study we only report mosaicism results for samples with *SRY* KI, given that *SRY* negative embryos were not analyzed for mosaicism.

High rates of mosaicism has been reported for zygote gene editing in cattle, sheep and pigs^25–27^. Several strategies were proposed to reduce mosaicism^24^ including altering the concentration of editing reagents, the form of Cas9, and the time of injection, although no approach has eliminated mosaic mutations resulting from CRISPR/Cas9 genome editing of embryos. Ultimately, the efficiency of the gRNA and repair machinery to introduce mutations prior to DNA replication in zygotes may be the most important factor to reduce mosaicism.

Although we were able to produce non-mosaic gene KI bovine embryos, the ultimate goal is to reliably and efficiently produce live animals. Here we have concluded further optimization needs to be undertaken to increase the efficiency of producing viable, non-mosaic gene KI embryos.

Electroporation offers a promising approach to deliver editing reagents to zygotes^28,29^, although to date efficiencies of HDR editing in electroporated bovine embryos are low^30^. The majority of KI animals created through electroporation of zygotes use single-stranded oligodeoxynucleotides (ssODN) donors, which have been shown to be stable and efficient in being incorporated into the genome through homology directed repair (HDR)^31^. Unfortunately, the size of the ssODN donor is a limitation as optimal ssODN donors are limited to a only about 100 base pairs^32^. This limits donor repair templates to < 1 kb when electroporating ssODN^33^, although large insertions have been achieved in zygotes when using a ssODN-mediated KI approach in combination with microinjection of large donor repair templates^34^.

Studies in mice have been able to overcome the size limitation of ssODN donors for embryo KI by using adeno-associated virus (AAV) to deliver the repair constructs in combination with CRISPR/Cas9 microinjection^35^ and to transfect large HDR donors of up to 4.9 kb prior to electroporation^36^. Although this approach has yet to be applied to livesetock^33^, it has very high embryo survival rates with editing reported in up to 100% of offspring^37^.

While there are still improvements to be made, this study optimized approaches to facilitate the one-step introduction of a gene construct at a specific location in the bovine genome, resulting in high KI efficiency and reduced mosaicism. Based on PCR using primers outside homology regions linking to the HMEJ repair template we demonstrated that 37% KI embryos were generated, with the 1.8 kb *SRY* gene construct correctly introduced into the target locus. Clonal sequences generated from PacBio revealed that 36.9% of these KI embryos were non-mosaic. This HMEJ-approach could be used to facilitate the introduction of novel genes or useful genetic variants into livestock breeding programs.

## Materials and methods

### Experimental design

This study was designed to compare the development and KI rates following microinjection with a gRNA/Cas9 RNP complex and either a HMEJ donor template or a HR donor template into 25 groups of 30 MII oocyte, or eight groups of 30 presumptive zygotes 6hpi. The results from each of these four groups were compiled and utilized to make three fundamental comparisons: (1) the percentage of microinjected embryos that reached the blastocyst stage as compared to non-injected controls; (2) the KI efficiency and percentage of non-mosaic blastocysts when comparing the HMEJ donor template to the HR donor template; and (3) the KI efficiency and percentage of non-mosaic blastocysts when comparing the HMEJ donor template injected into MII oocytes or presumptive zygotes 6 hpi.

### Animal care

All experiments involving animals were approved and performed in compliance with the Institutional Animal Care and Use protocol #20595 at the University of California, Davis. Recipient cattle were maintained at the University of California, Davis Beef Barn.

### Embryo production

Bovine ovaries were collected from a local slaughterhouse and transported to the laboratory at 35–37 °C in sterile saline. Cumulus-oocyte-complexes (COCs) were aspirated from follicles and groups of 50 COCs were transferred to 4-well dishes containing 400 μL of maturation media^38^. COCs were incubated for 18 h at 38.5 °C in a humidified 5% CO_2_ incubator. Approximately 25 oocytes per drop were fertilized in 60 μL drops of SOF-IVF^38^ with 2 × 10^6^ sperm per mL and incubated for 6 h at 38.5 °C in a humidified 5% CO_2_ incubator. Presumptive zygotes were denuded by light vortex in SOF-HEPES medium^38^ for 5 min. 25 zygotes per drop were incubated in 50 μL drops of KSOM culture media (Evolve, Zenith Biotech) at 38.5 °C in a humidified atmosphere of 5% CO_2_, 5% O_2_, and 90% N_2_ for 7–8 days.

### Guide-RNA design and testing

Guide-RNAs were designed and tested as previous described^11^. In short, guides sequences were designed targeting the X-chromosome, 10 kb downstream of the *ZFX* gene, with no less than three mismatches in the guide sequence for off-target sites and at least one mismatch in the seed region (8–11 bp upstream of the PAM sequence) when compared to the bovine reference genome. Mutation rate for each guide was determined by laser-assisted cytoplasmic injection^20^ of in vitro fertilized embryos with 6 pL of a solution containing 67 ng/μL of in vitro transcribed gRNA alongside 167 ng/μL of Cas9 protein (PNA Bio) incubated at room temperature for 30 min prior to injection. Embryos that reached blastocyst stage were lysed in 10 μL of Epicenter DNA extraction buffer (Lucigen) using a thermal cycler at 65 °C for 6 min, 98 °C for 2 min and held at 4 °C. The target region was amplified by two rounds of the polymerase chain reaction (PCR) using primers developed using Primer3 (Supplementary Information, Table S4)^39,40^. The first round of PCR was performed on a thermal cycler with 10 μL GoTaq Green Master Mix (Promega), 0.4 μL of each primer at 10 mM and 9.2 μL of DNA in lysis buffer for 5 min at 95 °C, 35 cycles of 30 s at 95 °C, 30 s at anneal temp (Supplementary Information, Table S4), and 30 s at 72 °C, followed by 5 min at 72 °C. The second round of PCR was run with 10 μL GoTaq Green Master Mix (Promega), 4.2 μL of water, 0.4 μL of each primer at 10 mM and 5 μL of first round PCR for 3 min at 95 °C, 35 cycles of 30 s at 95 °C, 30 s at anneal temp (Supplementary Information, Table S4), and 30 s at 72 °C, followed by 5 min at 72 °C. Products were visualized on a 1% agarose gel using a gel imager, purified using the QIAquick Gel Extraction Kit (Qiagen) and Sanger sequenced (GeneWiz).

### Donor plasmid construction

Donor plasmids were created to introduce *SRY* into the ZFX locus on the X-chromosome 10 kb downstream of the *ZFX* gene. The donor plasmids were commercially synthesized (GeneWiz) to contain the endogenous *Bos taurus SRY* promoter and coding sequence (Accession U15569)^41^. 1 kb homology arms were commercially synthesized (GeneWiz) containing regions flanking the cut site and inserted into the donor plasmids using Gibson Assembly Master Mix (NEB), with (hmejSRYp) and without (hrSRYp) the endogenous CRISPR target site flanking the homology arms (Fig. 2). Plasmids were clonally amplified using 5-alpha Chemically Competent *E. coli* (High Efficiency) (NEB) and extracted using the EndoFree Plasmid Maxi Kit (Qiagen).

### Cytoplasmic injection and PCR amplification for KI efficiency

KI of donor plasmids was attempted using laser-assisted cytoplasmic injection^20^ of in vitro matured oocytes after 18 h of maturation and in vitro fertilized embryos 6 hpi with 6 pL of a solution containing 67 ng/μL of in vitro transcribed gRNA, 167 ng/μL of Cas9 protein (PNA Bio) and 133 ng/μL of donor plasmid. Injected MII oocytes were subsequently co-cultured with cumulus-oocyte-complexes (COCs) and in vitro fertilized following procedures previously described for sheep^26^. Embryos were scored for developmental stage reached at day 7–8. Embryos that reached blastocyst stage were lysed as described above and underwent whole-genome amplification using the Repli-G Mini kit (Qiagen). Target regions were amplified using primers developed using Primer3 (Supplementary Information, Fig. S1 and Table S4)^39,40^. PCR was performed on a thermal cycler with 12.5 μL LongAmp *Taq* 2X Master Mix (NEB), 9.5 μL of H_2_O, 1 μL of each primer at 10 mM and 1 μL of DNA for 5 min at 94 °C, 35 cycles of 30 s at 94 °C, 30 s at anneal temp (Supplementary Information, Table S4) and 4 min at 65 °C, followed by 15 min at 65 °C. Products were visualized on a 1% agarose gel using a gel imager, purified using the QIAquick Gel Extraction Kit (Qiagen) and Sanger sequenced (GeneWiz).

### Next-generation sequencing of knock-in samples

Samples that were positive for *SRY* KI were PCR amplified using a dual round PCR approach described above to barcode samples for pooled sequencing (Supplemental File S2) with the use of 5 cycles in the first round of PCR and 35 cycles in the second round of PCR. Barcoded amplicons underwent SMRTbell library preparation and were sequenced on a PacBio Sequel II sequencer (GeneWiz). Consensus sequences were called, reads separated by barcode and BAM converted to individual FASTQ files using SMRT Link v8.0.0.80529 (https://www.pacb.com/support/software-downloads/). Raw reads were aligned to each target site using Bowtie2-default v2.3.4.1^42^ (Supplementary Information, Fig. S2). Alignments were visualized using Integrative Genomics Viewer v2.6.2^43^. For mosaic analysis, reads were aligned to each target site using BWA v0.7.16a^44^. SAM files were converted to BAM files, sorted and indexed using SAMtools v1.9^45^. Number and types of alleles were determined for each sample using CrispRVariants v1.12.0^46^.

### Embryo biopsy and vitrification

Biopsies were performed seven days post-fertilization on all embryos that reached the blastocyst stage as previously described^47^. In short, small 8–10 cell biopsies were taken from the trophectoderm of expanded blastocysts using a microblade in Dulbecco’s phosphate-buffered saline lacking magnesium chloride or calcium chloride. Biopsies were transferred to 10 μL of Epicenter DNA extraction buffer (Lucigen) and lysed as described above. Blastocysts were returned to culture media for two hours to monitor re-expansion. Re-expanded blastocysts were non-surgically transferred to synchronized recipients, as described below, or vitrified for transfer at a later date. Blastocysts were vitrified using Vit Kit-Freeze (Irvine Scientific). Embryos were placed in ES solution until re-expanded, followed by 2 min in VS solution before being transferred to a CryoTip straw (Irvine Scientific). The straw was the sealed and plunged into liquid nitrogen prior to transfer to a Dewar for storage. Embryos were thawed for transfer using Vit Kit-Thaw (Irvine Scientific). Straws were thawed in a 37 °C water bath for 3 s, followed by transfer of the embryo to TS solution for 1 min. The embryo was transferred to DS solution for 4 min, then washed twice in WS solution prior to loading the embryo into the straw for transfer.

### Embryo transfer

Recipient estrus synchronization was initiated by inserting an intravaginal progesterone device (1.38 g; Eazi-Breed CIDR; Zoetis) and intramuscular administration of gonadotropin (100 mcg; Factrel; Zoetis) on day 0 (sixteen days prior to transfer). On day 7, the CIDR was removed and intramuscular prostaglandin (25 mg; Lutalyse; Zoetis) was administered. Recipients were monitored for estrus, and a second intramuscular dose of gonadotropin (100 mcg; Factrel; Zoetis) was administered on day 9. Prior to transfer on day 16, recipient response to synchronization was confirmed via detection of an appropriate corpus luteum with transrectal ultrasonography. Prior to transfer, each recipient received a caudal epidural using 100 mg 2% lidocaine (Xylocaine; Fresenius). Embryos were transferred via non-surgical, transcervical technique, and the blastocyst was deposited into the uterine horn ipsilateral to the corpus luteum. Pregnancy was diagnosed on day 35 of embryonic development by transrectal ultrasonography (5.0 MHz linear probe; EVO Ibex, E.I. Medical Imaging).

### Statistical analysis

Comparison between development for guide analysis and mutation rates were evaluated using a linear model and statistical significance was determined using a Chi-square test. To analyze the level of mosaicism, an ANOVA test was used to determine significance between number of alleles per sample when injecting MII oocytes compared to 6 hpi. For KI evaluation, injected groups or non-injected controls were blocked by replicate to determine the significance between the means in R using the Tukey method. A generalized linear model was run for samples to determine the effects of sex, timing and donor plasmid on mutation rates and KI efficiency. Each blastocyst was considered an individual test, evaluated using a binomial distribution and analyzed using a linear regression model. Once the unimportant variables were excluded, the groups were blocked by type and a two by two Chi square test was used to determine significance. Differences were considered significant when *P* < 0.05.

## Supplementary information

Supplementary information.

## Data Availability

Raw sequence reads from PacBio Sequel II and Illumina MiSeq sequencing are available in the NCBI Sequence Read Archive as BioProject PRJNA635115 and SRA accession number SRR11850981. Individual results for the blastocyst development and mutation rate from each replicate (~ 30 embryos) of control and microinjected embryos are available in Supplementary Table S5–S6.
