# Supplementary material for: Harnessing endogenous repair mechanisms for targeted gene knock-in of bovine embryos
Source: Sci Rep. 2020 Sep 29;10:16031. doi: 10.1038/s41598-020-72902-x (PMC7525238; doi:10.1038/s41598-020-72902-x)
Supplement: Supplementary file 1 — Supplementary information. [file 41598_2020_72902_MOESM1_ESM.docx]

**Supplemental Information**

Harnessing endogenous repair mechanisms for targeted gene knock-in of bovine embryos

Joseph R. Owen^#1^, Sadie L. Hennig^#1^, Bret R. McNabb^2^, Jason C. Lin^1^, Amy E. Young^1^, Pablo J. Ross^1^, James D. Murray^1,2^, and Alison L. Van Eenennaam^1^

^1^Department of Animal Science, University of California – Davis, Davis, CA, United States

^2^Department of Population Health and Reproduction, School of Veterinary Medicine, University of California – Davis, Davis, CA, USA

^#^Joseph R. Owen and Sadie L. Hennig contributed equally.

Corresponding Author email: alvaneenennaam@ucdavis.edu

**Supplementary Table S1.** Mutation rate in embryos for each guide analyzed using PCR and Sanger sequencing. Multiple guides were tested targeting each locus to obtain highest efficiency guide. Letters that differ in the same column are significantly different (P < 0.05). Each chromosome independently tested using a two-by-two χ^2^ test.

| **Allele** | **gRNA** | **Total Embryos** | **% Blastocysts (n)** | **Blastocysts Analyzed** | **% Mutation Rate (n)** |
| --- | --- | --- | --- | --- | --- |
| Control | - | 120 | 27 (32)^a^ | - | - |
| ZFX | 1 | 62 | 26 (16)^a^ | 16 | 38 (6)^a^ |
|  | 2 | 80 | 18 (14)^b^ | 14 | 57 (8)^a^ |
|  | 3 | 94 | 15 (14)^b^ | 11 | 82 (9)^b^ |

**Supplementary Table S2.** Number of alleles and percentage of each corresponding allele per sample detected at the cut-site of Cas9 protein injected embryos. WT = percentage of reads that were wild type sequence. SRY = insertion of the sex-determining region Y gene into the target location. Alleles 1-4 are percent reads with each of the alleles containing insertion or deletions found in the samples. Bold samples were non-mosaic and contained no wild type sequence.

| **Time of Injection** | **Donor** | **Sample** | **Sex** | **# of alleles** | **% of Reads for Each Allele** | | | | | |
| --- | --- | --- | --- | --- | --- | --- | --- | --- | --- | --- |
|  |  |  |  |  | **WT** | **SRY** | **Allele 1** | **Allele 2** | **Allele 3** | **Allele 4** |
| MII oocytes | HMEJ | **1** | **male** | **1** | **-** | **100** | **-** | **-** | **-** | **-** |
|  |  | **2** | **female** | **1** | **-** | **100** | **-** | **-** | **-** | **-** |
|  |  | 3 | male | 5 | 34 | 14 | 37 | 11 | 4 | - |
|  |  | 4 | female | 6 | 37 | 8 | 31 | 17 | 4 | 3 |
|  |  | **5** | **male** | **1** | **-** | **100** | **-** | **-** | **-** | **-** |
|  |  | 6 | female | 6 | 23 | 13 | 28 | 22 | 13 | 2 |
|  |  | **7** | **female** | **1** | **-** | **100** | **-** | **-** | **-** | **-** |
|  |  | 8 | female | 6 | 31 | 8 | 41 | 12 | 5 | 3 |
|  |  | **9** | **female** | **1** | **-** | **100** | **-** | **-** | **-** | **-** |
|  |  | 10 | female | 5 | 5 | 21 | 64 | 7 | 3 | - |
|  |  | 11 | male | 6 | 47 | 8 | 19 | 12 | 9 | 5 |
|  |  | 12 | female | 6 | 37 | 8 | 23 | 12 | 12 | 9 |
|  |  | 13 | male | 6 | 30 | 9 | 22 | 19 | 10 | 10 |
|  |  | 14 | male | 4 | - | 10 | 54 | 18 | 18 | - |
|  |  | 15 | female | 4 | 33 | 55 | 7 | 5 | - | - |
|  |  | 16 | male | 4 | - | 9 | 54 | 21 | 17 | - |
|  |  | 17 | female | 4 | - | 9 | 56 | 18 | 17 | - |
|  |  | 18 | female | 4 | - | 9 | 55 | 18 | 18 | - |
|  |  | 19 | male | 4 | 21 | 9 | 66 | 5 | - | - |
|  |  | 20 | female | 6 | 18 | 9 | 57 | 11 | 3 | 3 |
|  |  | 21 | female | 3 | 19 | 8 | 74 | - | - | - |
|  |  | **22** | **male** | **1** | **-** | **100** | **-** | **-** | **-** | **-** |
|  |  | **23** | **male** | **1** | **-** | **100** | **-** | **-** | **-** | **-** |
|  |  | 24 | male | 5 | 4 | 71 | 9 | 14 | 6 | - |

| **Time of Injection** | **Donor** | **Sample** | **Sex** | **# of alleles** | **% of Reads for Each Allele** | | | | | |
| --- | --- | --- | --- | --- | --- | --- | --- | --- | --- | --- |
|  |  |  |  |  | **WT** | **SRY** | **Allele 3** | **Allele 4** | **Allele 5** | **Allele 6** |
| MII oocytes | HMEJ | **25** | **male** | **1** | **-** | **100** | **-** | **-** | **-** | **-** |
|  |  | 26 | male | 6 | 30 | 10 | 26 | 22 | 9 | 4 |
|  |  | 27 | female | 6 | 36 | 9 | 40 | 8 | 5 | 2 |
|  |  | 28 | male | 6 | 29 | 10 | 46 | 10 | 3 | 2 |
|  |  | 29 | male | 6 | 38 | 9 | 37 | 9 | 4 | 4 |
|  |  | 30 | female | 2 | - | 35 | 65 | - | - | - |
|  |  | 31 | female | 2 | - | 33 | 68 | - | - | - |
|  |  | **32** | **male** | **1** | **-** | **100** | **-** | **-** | **-** | **-** |
|  |  | 33 | female | 4 | 27 | 9 | 50 | 14 | - | - |
|  |  | 34 | male | 3 | - | 8 | 50 | 42 | - | - |
|  |  | **35** | **male** | **1** | **-** | **100** | **-** | **-** | **-** | **-** |
|  |  | 36 | female | 3 | - | 8 | 50 | 43 | - | - |
|  |  | 37 | female | 6 | 20 | 23 | 30 | 18 | 5 | 5 |
|  |  | 38 | male | 3 | 21 | 77 | 2 | - | - | - |
|  |  | 39 | male | 5 | 20 | 9 | 54 | 13 | 4 | - |
|  |  | 40 | male | 4 | 68 | 10 | 18 | 3 | - | - |
|  |  | 41 | male | 4 | 66 | 9 | 21 | 5 | - | - |
|  |  | **42** | **male** | **1** | **-** | **100** | **-** | **-** | **-** | **-** |
|  |  | 43 | female | 2 | - | 10 | 90 | - | - | - |
|  |  | **44** | **male** | **1** | **-** | **100** | **-** | **-** | **-** | **-** |
|  |  | **45** | **female** | **1** | **-** | **100** | **-** | **-** | **-** | **-** |
|  |  | **46** | **male** | **1** | **-** | **100** | **-** | **-** | **-** | **-** |
|  |  | **47** | **male** | **1** | **-** | **100** | **-** | **-** | **-** | **-** |
|  |  | **48** | **male** | **1** | **-** | **100** | **-** | **-** | **-** | **-** |
|  |  | **49** | **male** | **1** | **-** | **100** | **-** | **-** | **-** | **-** |
|  |  | **50** | **male** | **1** | **-** | **100** | **-** | **-** | **-** | **-** |
|  |  | 51 | female | 2 | - | 11 | 89 | - | - | - |
|  |  | **52** | **female** | **1** | **-** | **100** | **-** | **-** | **-** | **-** |
| **Time of Injection** | **Donor** | **Sample** | **Sex** | **# of alleles** | **% of Reads for Each Allele** | | | | | |
|  |  |  |  |  | **WT** | **SRY** | **Allele 3** | **Allele 4** | **Allele 5** | **Allele 6** |
| MII | HMEJ | 53 | female | 5 | 19 | 9 | 55 | 13 | 4 | - |
|  |  | 54 | female | 2 | - | 9 | 91 | - | - | - |
|  |  | **55** | **female** | **1** | **-** | **100** | **-** | **-** | **-** | **-** |
|  |  | 56 | male | 2 | - | 9 | 92 | - | - | - |
|  |  | 57 | female | 6 | 53 | 11 | 15 | 9 | 7 | 6 |
|  |  | 58 | female | 6 | 54 | 9 | 15 | 10 | 7 | 6 |
|  |  | 59 | female | 5 | 19 | 8 | 56 | 15 | 2 | - |
|  |  | **60** | **male** | **1** | **-** | **100** | **-** | **-** | **-** | **-** |
|  |  | 61 | female | 6 | 37 | 13 | 28 | 11 | 6 | 6 |
|  |  | 62 | male | 3 | 77 | 14 | 8 | - | - | - |
|  |  | 63 | female | 6 | 32 | 8 | 37 | 8 | 8 | 7 |
|  |  | **64** | **female** | **1** | **-** | **100** | **-** | **-** | **-** | **-** |
|  |  | **65** | **male** | **1** | **-** | **100** | **-** | **-** | **-** | **-** |
|  |  | **66** | **female** | **1** | **-** | **100** | **-** | **-** | **-** | **-** |
|  |  | 67 | male | 6 | 29 | 8 | 38 | 13 | 6 | 5 |
|  |  | 68 | male | 6 | 32 | 9 | 36 | 12 | 6 | 5 |
|  |  | 69 | male | 3 | 12 | 9 | 79 | - | - | - |
|  |  | **70** | **male** | **1** | **-** | **100** | **-** | **-** | **-** | **-** |
|  |  | 71 | male | 4 | 86 | 7 | 4 | 3 | - | - |
|  |  | 72 | female | 4 | 83 | 6 | 6 | 5 | - | - |
|  | HR | **1** | female | **1** | **-** | **100** | **-** | **-** | **-** | **-** |
|  |  | **2** | male | **1** | **-** | **100** | **-** | **-** | **-** | **-** |
|  |  | 3 | female | 4 | 83 | 7 | 6 | 4 | - | - |
|  |  | 4 | female | 2 | - | 8 | 92 | - | - | - |
|  |  | 5 | female | 2 | - | 8 | 92 | - | - | - |
| 6hpi | HMEJ | **1** | **male** | **1** | **-** | **100** | **-** | **-** | **-** | **-** |
|  |  | 2 | female | 5 | 57 | 12 | 21 | 7 | 3 | - |
|  |  | 3 | female | 5 | 58 | 13 | 20 | 6 | 3 | - |
|  |  | **4** | **male** | **1** | **-** | **100** | **-** | **-** | **-** | **-** |
| **Time of Injection** | **Donor** | **Sample** | **Sex** | **# of alleles** | **% of Reads for Each Allele** | | | | | |
|  |  |  |  |  | **WT** | **SRY** | **Allele 3** | **Allele 4** | **Allele 5** | **Allele 6** |
| 6hpi | HMEJ | **5** | **female** | **1** | **-** | **100** | **-** | **-** | **-** | **-** |
|  |  | **6** | **female** | **1** | **-** | **100** | **-** | **-** | **-** | **-** |
|  |  | 7 | female | 2 | - | 11 | 89 | - | - | - |
|  |  | **8** | **male** | **1** | **-** | **100** | **-** | **-** | **-** | **-** |
|  |  | 9 | male | 4 | 53 | 8 | 32 | 8 | - | - |
|  |  | **10** | **male** | **1** | **-** | **100** | **-** | **-** | **-** | **-** |
|  |  | 11 | female | 5 | 7 | 8 | 73 | 8 | 5 | - |
|  |  | 12 | male | 5 | - | 9 | 49 | 21 | 16 | 6 |
|  | HR | 1 | female | 5 | 62 | 9 | 16 | 10 | 3 | - |
|  |  | 2 | female | 5 | 18 | 8 | 62 | 7 | 5 | - |
|  |  | 3 | female | 6 | 18 | 18 | 46 | 9 | 5 | 5 |

**Supplementary Table S3.** Results from embryo transfer of MII injected oocytes followed by *in vitro* fertilization. Twelve of the 18 transferred blasts from trial two were vitrified, while six were fresh day eight blastocysts.

| **Trial** | **Date** | **Biopsied** | **Vitrified** | **Blasts Transferred** | **Pregnancy** |
| --- | --- | --- | --- | --- | --- |
| 1 | 9/28/2018 | yes | no | 10 | 0 |
| 2 | 12/21/2018 | yes | yes | 12 | 0 |
|  |  |  | no | 6 | 0 |

**Supplementary Table S4.** Sequence of primers used for PCR amplification of target region, evaluation of *SRY* knock-in, barcodes used for sequencing and guide-RNA sequences.

|  | **Name** | **Sequence 5’- 3’** | **T_m_ (^o^C)** |
| --- | --- | --- | --- |
| On-Target PCR primers | ZFXgF | TCCAAGGAGCTATGTCACAGAA | 60.8 |
|  | ZFXgR | CACTAGCTTTGGGCGATATGA | 60.8 |
|  | ZFXknF | CCGCTTCAAATCAGTTTAATCC | 58.9 |
|  | ZFXknR | CCCCACCAGGAAAGTACAAA | 60.4 |
|  | SRYknF | TGGTCCTCTGTTAATCAGTTCTTTC | 61.3 |
|  | SRYknR | GGAACTGCTTGGGTACCAAG | 62.4 |
|  | DDX3-1F^47^ | AGGAAGCCAGGAAAGTAA | 55.3 |
|  | DDX3-1R^47^ | CATCCACGTTCTAAGTCTC | 58.0 |
| Barcode Primers | BC1F | TCAGACGATGCGTCATAGATCTCTCGAGGTT | 62.0 |
|  | BC1R | TCAGACGATGCGTCATGTAGTCGAATTCGTT | 62.0 |
|  | BC17F | CATAGCGACTATCGTGAGATCTCTCGAGGTT | 62.0 |
|  | BC17R | CATAGCGACTATCGTGGTAGTCGAATTCGTT | 62.0 |
|  | BC29F | GCTCGACTGTGAGAGAAGATCTCTCGAGGTT | 62.0 |
|  | BC29R | GCTCGACTGTGAGAGAGTAGTCGAATTCGTT | 62.0 |
|  | BC34F | ACTCTCGCTCTGTAGAAGATCTCTCGAGGTT | 62.0 |
|  | BC34R | ACTCTCGCTCTGTAGAGTAGTCGAATTCGTT | 62.0 |
|  | BC38F | TGCTCGCAGTATCACAAGATCTCTCGAGGTT | 62.0 |
|  | BC38R | TGCTCGCAGTATCACAGTAGTCGAATTCGTT | 62.0 |
|  | BC40F | CAGTGAGAGCGCGATAAGATCTCTCGAGGTT | 62.0 |
|  | BC40R | CAGTGAGAGCGCGATAGTAGTCGAATTCGTT | 62.0 |
|  | BC48F | TCACACTCTAGAGCGAAGATCTCTCGAGGTT | 62.0 |
|  | BC48R | TCACACTCTAGAGCGAGTAGTCGAATTCGTT | 62.0 |
|  | BC52F | GCAGACTCTCACACGCAGATCTCTCGAGGTT | 62.0 |
|  | BC52R | GCAGACTCTCACACGCGTAGTCGAATTCGTT | 62.0 |
|  | BC54F | GTGTGAGATATATATCAGATCTCTCGAGGTT | 62.0 |
|  | BC54R | GTGTGAGATATATATCGTAGTCGAATTCGTT | 62.0 |
|  | BC62F | GACAGCATCTGCGCTCAGATCTCTCGAGGTT | 62.0 |
|  | BC62R | GACAGCATCTGCGCTCGTAGTCGAATTCGTT | 62.0 |
|  | BC70F | CTGCGCAGTACGTGCAAGATCTCTCGAGGTT | 62.0 |
|  | BC70R | CTGCGCAGTACGTGCAGTAGTCGAATTCGTT | 62.0 |
|  | BC9F | CTGCGTGCTCTACGACAGATCTCTCGAGGTT | 62.0 |
|  | BC9R | CTGCGTGCTCTACGACGTAGTCGAATTCGTT | 62.0 |
| Guide RNA | ZFXg1 | ACAACCCAAAATGAAGGGGG | - |
|  | ZFXg2 | AATACAACCCAAAATGAAGG | - |
|  | ZFXg3 | CTCCCATGTCATAACTTCTG | - |

**Supplementary Table S5.** Development results from each collection by donor vector injected (hmejSRYp or hrSRYp), time of injection (MII oocytes or 6 hours post insemination (6hpi)), sex of the blastocysts, number of blastocysts collected, total number of embryos injected or cultured and resulting percentage of embryos that developed to the blastocyst stage.

| **Collection** | **Donor** | **Time of Injection** | **Sex** | **Blast (n)** | **Total Embryos** | **Blast (%)** |
| --- | --- | --- | --- | --- | --- | --- |
| 1 | control | control | control | 25 | 80 | 31.3 |
| 1 | HMEJ | MII | male | 2 | 20 | 10.0 |
| 2 | control | control | control | 24 | 80 | 30.0 |
| 2 | HMEJ | MII | female | 2 | 20 | 10.0 |
| 2 | HMEJ | MII | male | 2 | 20 | 10.0 |
| 3 | control | control | control | 26 | 80 | 32.5 |
| 3 | HMEJ | MII | female | 1 | 10 | 10.0 |
| 3 | HMEJ | MII | male | 1 | 10 | 10.0 |
| 4 | control | control | control | 23 | 80 | 38.8 |
| 4 | HMEJ | MII | male | 3 | 25 | 12.0 |
| 5 | control | control | control | 26 | 80 | 32.5 |
| 5 | HMEJ | MII | female | 7 | 100 | 7.0 |
| 5 | HMEJ | MII | male | 13 | 100 | 13.0 |
| 6 | control | control | control | 22 | 80 | 27.5 |
| 6 | HMEJ | MII | female | 5 | 50 | 10.0 |
| 6 | HMEJ | MII | male | 8 | 50 | 16.0 |
| 7 | control | control | control | 23 | 80 | 28.8 |
| 7 | HMEJ | MII | female | 12 | 100 | 12.0 |
| 7 | HMEJ | MII | male | 9 | 100 | 9.0 |
| 8 | control | control | control | 27 | 80 | 33.8 |
| 8 | HMEJ | MII | female | 2 | 20 | 10.0 |
| 8 | HMEJ | MII | male | 3 | 20 | 15.0 |
| 9 | control | control | control | 19 | 80 | 23.8 |
| 9 | HMEJ | MII | female | 3 | 100 | 3.0 |
| 9 | HMEJ | MII | male | 16 | 100 | 16.0 |
| 10 | control | control | control | 24 | 80 | 30.0 |
| 10 | HMEJ | MII | female | 9 | 40 | 22.5 |
| 10 | HMEJ | MII | male | 1 | 40 | 2.5 |
| 11 | control | control | control | 23 | 80 | 28.8 |
| 11 | HMEJ | MII | female | 13 | 100 | 13.0 |
| 11 | HMEJ | MII | male | 7 | 100 | 7.0 |
| 12 | control | control | control | 24 | 80 | 30.0 |
| 12 | HMEJ | MII | female | 5 | 50 | 10.0 |
| 12 | HMEJ | MII | male | 7 | 50 | 14.0 |
| 13 | control | control | control | 25 | 80 | 31.3 |
| 13 | HMEJ | MII | female | 2 | 40 | 5.0 |
| 13 | HMEJ | MII | male | 7 | 40 | 17.5 |
| 14 | control | control | control | 22 | 80 | 27.5 |
| 14 | HMEJ | MII | female | 4 | 100 | 4.0 |
| 14 | HMEJ | MII | male | 15 | 100 | 15.0 |
| 15 | control | control | control | 29 | 80 | 36.3 |
| 15 | HR | MII | female | 3 | 25 | 12.0 |
| 15 | HR | MII | male | 4 | 25 | 16.0 |
| 16 | control | control | control | 26 | 80 | 32.5 |
| 16 | HR | MII | female | 5 | 40 | 12.5 |
| 16 | HR | MII | male | 3 | 40 | 7.5 |
| 17 | control | control | control | 20 | 80 | 25.0 |
| 17 | HR | MII | female | 2 | 30 | 6.7 |
| 17 | HR | MII | male | 6 | 30 | 20.0 |
| 18 | control | control | control | 22 | 80 | 27.5 |
| 18 | HR | MII | female | 5 | 30 | 16.7 |
| 18 | HMEJ | 6hpi | female | 2 | 15 | 13.3 |
| 18 | HR | 6hpi | female | 5 | 15 | 33.3 |
| 18 | HR | MII | male | 3 | 30 | 10.0 |
| 18 | HMEJ | 6hpi | male | 2 | 15 | 13.3 |
| 18 | HR | 6hpi | male | 2 | 15 | 13.3 |
| 19 | control | control | control | 21 | 80 | 26.3 |
| 19 | HMEJ | 6hpi | female | 3 | 20 | 15.0 |
| 19 | HR | 6hpi | female | 1 | 20 | 5.0 |
| 19 | HMEJ | 6hpi | male | 6 | 20 | 30.0 |
| 19 | HR | 6hpi | male | 6 | 20 | 30.0 |
| 20 | control | control | control | 24 | 80 | 30.0 |
| 20 | HR | 6hpi | female | 5 | 30 | 16.7 |
| 21 | control | control | control | 23 | 80 | 28.8 |
| 21 | HR | 6hpi | female | 3 | 15 | 20.0 |
| 21 | HR | 6hpi | male | 3 | 15 | 20.0 |
| 22 | control | control | control | 22 | 80 | 27.5 |
| 22 | HR | 6hpi | female | 2 | 15 | 13.3 |
| 22 | HR | 6hpi | male | 1 | 15 | 6.7 |
| 23 | control | control | control | 24 | 80 | 30.0 |
| 23 | HMEJ | MII | male | 3 | 30 | 10.0 |
| 24 | control | control | control | 23 | 80 | 28.8 |
| 24 | HMEJ | MII | female | 4 | 25 | 16.0 |
| 24 | HMEJ | MII | male | 2 | 25 | 8.0 |
| 25 | control | control | control | 22 | 80 | 27.5 |
| 25 | HMEJ | MII | female | 1 | 30 | 3.3 |
| 25 | HMEJ | MII | male | 6 | 30 | 20.0 |
| 26 | control | control | control | 21 | 80 | 26.3 |
| 26 | HMEJ | 6hpi | female | 8 | 50 | 16.0 |
| 26 | HMEJ | 6hpi | male | 9 | 50 | 18.0 |
| 27 | control | control | control | 20 | 80 | 25.0 |
| 27 | HMEJ | MII | female | 4 | 30 | 13.3 |
| 27 | HMEJ | MII | male | 3 | 30 | 10.0 |
| 28 | control | control | control | 21 | 80 | 26.3 |
| 28 | HMEJ | MII | female | 1 | 15 | 6.7 |
| 28 | HMEJ | MII | male | 1 | 15 | 6.7 |
| 29 | control | control | control | 27 | 80 | 33.8 |
| 29 | HMEJ | MII | female | 11 | 100 | 11.0 |
| 29 | HMEJ | MII | male | 8 | 100 | 8.0 |
| 30 | control | control | control | 24 | 80 | 30.0 |
| 30 | HMEJ | MII | female | 3 | 30 | 10.0 |

**Supplementary Table S6.** Results for single blastocysts analyzed from each collected by donor vector injected (hmejSRYp or hrSRYp), time of injection (MII oocytes or 6 hours post insemination (6hpi), sex of the embryo, mutation in wild type allele, knock-in of SRY into the target location, type of knock-in (hemizygous, homozygous, homology independent target insertion (HII) or mosaic) and the barcodes used for PacBio sequencing if sample was positive for SRY knock-in. fail = samples that had no sexing PCR product were excluded from further analysis.

| **Collection** | **Sample** | **Donor** | **Time of Injection** | **Sex** | **Mutation** | **Knock-In** | **Knock-In Type** | **Forward Barcode** | **Reverse Barcode** |
| --- | --- | --- | --- | --- | --- | --- | --- | --- | --- |
| 1 | 1 | HMEJ | MII | male | yes | no | - | - | - |
| 1 | 2 | HMEJ | MII | male | yes | no | - | - | - |
| 2 | 1 | HMEJ | MII | male | yes | yes | hemi | BC1F | BC1R |
| 2 | 2 | HMEJ | MII | male | no | no | - | - | - |
| 2 | 3 | HMEJ | MII | female | yes | yes | homo | BC1F | BC17R |
| 2 | 4 | HMEJ | MII | female | yes | no | - | - | - |
| 3 | 1 | HMEJ | MII | male | no | no | - | - | - |
| 3 | 2 | HMEJ | MII | female | yes | no | - | - | - |
| 4 | 1 | HMEJ | MII | male | yes | yes | mosaic | BC1F | BC29R |
| 4 | 2 | HMEJ | MII | male | no | no | - | - | - |
| 4 | 3 | HMEJ | MII | male | yes | no | - | - | - |
| 5 | 1 | HMEJ | MII | female | yes | yes | mosaic | BC1F | BC34R |
| 5 | 2 | HMEJ | MII | male | yes | no | - | - | - |
| 5 | 3 | HMEJ | MII | male | yes | no | - | - | - |
| 6 | 1 | HMEJ | MII | female | yes | no | - | - | - |
| 6 | 2 | HMEJ | MII | male | yes | yes | HII | BC1F | BC38R |
| 6 | 3 | HMEJ | MII | female | yes | yes | mosaic | BC1F | BC40R |
| 6 | 4 | HMEJ | MII | male | yes | no | - | - | - |
| 7 | 1 | HMEJ | MII | male | no | no | - | - | - |
| 7 | 2 | HMEJ | MII | female | yes | no | - | - | - |
| 7 | 3 | HMEJ | MII | male | no | no | - | - | - |
| 7 | 4 | HMEJ | MII | female | no | no | - | - | - |
| 7 | 5 | HMEJ | MII | female | yes | yes | homo | BC1F | BC48R |
| 7 | 6 | HMEJ | MII | female | yes | yes | mosaic | BC1F | BC52R |
| 8 | 1 | HMEJ | MII | female | yes | yes | homo | BC1F | BC54R |
| 8 | 2 | HMEJ | MII | fail | - | - | - | - | - |
| 8 | 3 | HMEJ | MII | female | yes | yes | mosaic | BC1F | BC62R |
| 8 | 4 | HMEJ | MII | male | no | no | - | - | - |
| 8 | 5 | HMEJ | MII | male | yes | no | - | - | - |
| 8 | 6 | HMEJ | MII | male | no | no | - | - | - |
| 8 | 7 | HMEJ | MII | female | yes | no | - | - | - |
| 8 | 8 | HMEJ | MII | female | no | no | - | - | - |
| 9 | 1 | HMEJ | MII | fail | - | - | - | - | - |
| 9 | 2 | HMEJ | MII | fail | - | - | - | - | - |
| 9 | 3 | HMEJ | MII | male | yes | yes | mosaic | BC1F | BC70R |
| 9 | 4 | HMEJ | MII | fail | - | - | - | - | - |
| 9 | 5 | HMEJ | MII | fail | - | - | - | - | - |
| 9 | 6 | HMEJ | MII | fail | - | - | - | - | - |
| 9 | 7 | HMEJ | MII | female | yes | no | - | - | - |
| 9 | 8 | HMEJ | MII | fail | - | - | - | - | - |
| 10 | 1 | HMEJ | MII | male | no | no | - | - | - |
| 10 | 2 | HMEJ | MII | male | yes | no | - | - | - |
| 10 | 3 | HMEJ | MII | male | yes | no | - | - | - |
| 11 | 1 | HMEJ | MII | female | yes | yes | mosaic | BC1F | BC9R |
| 11 | 2 | HMEJ | MII | male | yes | no | - | - | - |
| 11 | 3 | HMEJ | MII | male | yes | no | - | - | - |
| 11 | 4 | HMEJ | MII | male | yes | yes | mosaic | BC17F | BC1R |
| 11 | 5 | HMEJ | MII | male | yes | no | - | - | - |
| 11 | 6 | HMEJ | MII | male | yes | yes | mosaic | BC17F | BC17R |
| 11 | 7 | HMEJ | MII | male | no | no | - | - | - |
| 12 | 1 | HMEJ | MII | female | yes | no | - | - | - |
| 12 | 2 | HMEJ | MII | fail | - | - | - | - | - |
| 12 | 3 | HMEJ | MII | female | no | no | - | - | - |
| 12 | 4 | HMEJ | MII | male | yes | no | - | - | - |
| 12 | 5 | HMEJ | MII | male | no | no | - | - | - |
| 12 | 6 | HMEJ | MII | female | yes | no | - | - | - |
| 12 | 7 | HMEJ | MII | male | yes | no | - | - | - |
| 12 | 8 | HMEJ | MII | male | no | no | - | - | - |
| 12 | 9 | HMEJ | MII | male | no | no | - | - | - |
| 12 | 10 | HMEJ | MII | female | no | no | - | - | - |
| 12 | 11 | HMEJ | MII | female | yes | yes | mosaic | BC17F | BC29R |
| 12 | 12 | HMEJ | MII | male | yes | yes | mosaic | BC17F | BC34R |
| 12 | 13 | HMEJ | MII | female | yes | yes | mosaic | BC17F | BC38R |
| 12 | 14 | HMEJ | MII | female | no | no | - | - | - |
| 12 | 15 | HMEJ | MII | female | yes | yes | mosaic | BC17F | BC40R |
| 13 | 1 | HMEJ | MII | male | yes | yes | mosaic | BC17F | BC48R |
| 13 | 2 | HMEJ | MII | female | yes | yes | mosaic | BC17F | BC52R |
| 13 | 3 | HMEJ | MII | female | yes | yes | mosaic | BC17F | BC54R |
| 13 | 4 | HMEJ | MII | female | yes | no | - | - | - |
| 13 | 5 | HMEJ | MII | male | yes | no | - | - | - |
| 13 | 6 | HMEJ | MII | male | no | no | - | - | - |
| 13 | 7 | HMEJ | MII | male | yes | yes | hemi | BC17F | BC62R |
| 13 | 8 | HMEJ | MII | male | yes | yes | hemi | BC17F | BC70R |
| 13 | 9 | HMEJ | MII | male | yes | yes | mosaic | BC17F | BC9R |
| 13 | 10 | HMEJ | MII | male | yes | yes | hemi | BC29F | BC1R |
| 13 | 11 | HMEJ | MII | male | yes | yes | mosaic | BC29F | BC17R |
| 13 | 12 | HMEJ | MII | female | yes | yes | mosaic | BC29F | BC29R |
| 13 | 13 | HMEJ | MII | male | yes | yes | mosaic | BC29F | BC34R |
| 13 | 14 | HMEJ | MII | female | yes | no | - | - | - |
| 13 | 15 | HMEJ | MII | fail | - | - | - | - | - |
| 13 | 16 | HMEJ | MII | female | yes | no | - | - | - |
| 13 | 17 | HMEJ | MII | male | yes | no | - | - | - |
| 13 | 18 | HMEJ | MII | male | yes | yes | mosaic | BC29F | BC38R |
| 14 | 1 | HMEJ | MII | fail | - | - | - | - | - |
| 14 | 2 | HMEJ | MII | fail | - | - | - | - | - |
| 14 | 3 | HMEJ | MII | fail | - | - | - | - | - |
| 14 | 4 | HMEJ | MII | female | no | no | - | - | - |
| 14 | 5 | HMEJ | MII | fail | - | - | - | - | - |
| 14 | 6 | HMEJ | MII | female | yes | no | - | - | - |
| 14 | 7 | HMEJ | MII | fail | - | - | - | - | - |
| 14 | 8 | HMEJ | MII | female | no | no | - | - | - |
| 14 | 9 | HMEJ | MII | fail | - | - | - | - | - |
| 15 | 1 | HMEJ | MII | male | no | no | - | - | - |
| 15 | 2 | HMEJ | MII | female | yes | yes | mosaic | BC29F | BC40R |
| 15 | 3 | HMEJ | MII | male | yes | no | - | - | - |
| 15 | 4 | HMEJ | MII | male | no | no | - | - | - |
| 15 | 5 | HMEJ | MII | female | yes | no | - | - | - |
| 15 | 6 | HMEJ | MII | male | yes | no | - | - | - |
| 15 | 7 | HMEJ | MII | male | yes | yes | mosaic | BC29F | BC48R |
| 15 | 8 | HMEJ | MII | female | yes | no | - | - | - |
| 15 | 9 | HMEJ | MII | male | no | no | - | - | - |
| 16 | 1 | HMEJ | MII | fail | - | - | - | - | - |
| 16 | 2 | HMEJ | MII | female | yes | no | - | - | - |
| 16 | 3 | HMEJ | MII | fail | - | - | - | - | - |
| 16 | 4 | HMEJ | MII | male | yes | yes | HII | BC29F | BC52R |
| 16 | 5 | HMEJ | MII | female | yes | no | - | - | - |
| 16 | 6 | HMEJ | MII | fail | - | - | - | - | - |
| 16 | 7 | HMEJ | MII | female | yes | no | - | - | - |
| 16 | 8 | HMEJ | MII | female | yes | no | - | - | - |
| 16 | 9 | HMEJ | MII | male | yes | no | - | - | - |
| 16 | 10 | HMEJ | MII | male | no | no | - | - | - |
| 16 | 11 | HMEJ | MII | female | yes | yes | mosaic | BC29F | BC54R |
| 16 | 12 | HMEJ | MII | male | yes | no | - | - | - |
| 16 | 13 | HMEJ | MII | female | yes | no | - | - | - |
| 16 | 14 | HMEJ | MII | male | yes | yes | mosaic | BC29F | BC62R |
| 16 | 15 | HMEJ | MII | female | yes | no | - | - | - |
| 16 | 16 | HMEJ | MII | female | yes | no | - | - | - |
| 16 | 17 | HMEJ | MII | female | yes | no | - | - | - |
| 16 | 18 | HMEJ | MII | male | yes | yes | hemi | BC29F | BC70R |
| 16 | 19 | HMEJ | MII | female | yes | yes | mosaic | BC29F | BC9R |
| 16 | 20 | HMEJ | MII | female | yes | yes | mosaic | BC34F | BC1R |
| 16 | 21 | HMEJ | MII | male | yes | yes | mosaic | BC34F | BC17R |
| 16 | 22 | HMEJ | MII | male | yes | yes | mosaic | BC34F | BC29R |
| 16 | 23 | HMEJ | MII | female | yes | no | - | - | - |
| 16 | 24 | HMEJ | MII | male | yes | no | - | - | - |
| 17 | 1 | HMEJ | MII | female | yes | no | - | - | - |
| 17 | 2 | HMEJ | MII | male | yes | yes | mosaic | BC34F | BC34R |
| 17 | 3 | HMEJ | MII | male | yes | no | - | - | - |
| 17 | 4 | HMEJ | MII | female | yes | no | - | - | - |
| 17 | 5 | HMEJ | MII | male | no | no | - | - | - |
| 18 | 1 | HMEJ | MII | male | yes | no | - | - | - |
| 18 | 2 | HMEJ | MII | fail | - | - | - | - | - |
| 18 | 3 | HMEJ | MII | male | yes | yes | mosaic | BC34F | BC38R |
| 18 | 4 | HMEJ | MII | male | no | no | - | - | - |
| 18 | 5 | HMEJ | MII | male | yes | no | - | - | - |
| 18 | 6 | HMEJ | MII | fail | - | - | - | - | - |
| 18 | 7 | HMEJ | MII | male | yes | yes | hemi | BC34F | BC40R |
| 18 | 8 | HMEJ | MII | female | yes | yes | mosaic | BC34F | BC48R |
| 18 | 9 | HMEJ | MII | male | yes | yes | HII | BC34F | BC52R |
| 18 | 10 | HMEJ | MII | female | yes | yes | HII | BC34F | BC54R |
| 18 | 11 | HMEJ | MII | male | yes | no | - | - | - |
| 18 | 12 | HMEJ | MII | male | no | no | - | - | - |
| 18 | 13 | HMEJ | MII | male | yes | yes | HII | BC34F | BC62R |
| 18 | 14 | HMEJ | MII | male | yes | no | - | - | - |
| 18 | 15 | HMEJ | MII | male | yes | no | - | - | - |
| 18 | 16 | HMEJ | MII | male | yes | yes | HII | BC34F | BC70R |
| 18 | 17 | HMEJ | MII | male | yes | no | - | - | - |
| 18 | 18 | HMEJ | MII | male | yes | yes | HII | BC34F | BC9R |
| 18 | 19 | HMEJ | MII | male | yes | yes | HII | BC38F | BC1R |
| 18 | 20 | HMEJ | MII | male | yes | yes | HII | BC38F | BC17R |
| 19 | 1 | HMEJ | MII | male | yes | no | - | - | - |
| 19 | 2 | HMEJ | MII | female | yes | yes | mosaic | BC38F | BC29R |
| 19 | 3 | HMEJ | MII | female | yes | yes | HII | BC38F | BC34R |
| 19 | 4 | HMEJ | MII | fail | - | - | - | - | - |
| 19 | 5 | HMEJ | MII | female | yes | yes | mosaic | BC38F | BC38R |
| 19 | 6 | HMEJ | MII | female | yes | yes | mosaic | BC38F | BC40R |
| 19 | 7 | HMEJ | MII | female | yes | yes | homo | BC38F | BC48R |
| 19 | 8 | HMEJ | MII | fail | - | - | - | - | - |
| 19 | 9 | HMEJ | MII | female | yes | yes | mosaic | BC38F | BC52R |
| 19 | 10 | HMEJ | MII | female | yes | yes | mosaic | BC38F | BC54R |
| 19 | 11 | HMEJ | MII | female | no | no | - | - | - |
| 19 | 12 | HMEJ | MII | female | no | no | - | - | - |
| 20 | 1 | HMEJ | MII | female | yes | yes | mosaic | BC38F | BC62R |
| 20 | 2 | HMEJ | MII | male | yes | no | - | - | - |
| 20 | 3 | HMEJ | MII | male | no | no | - | - | - |
| 20 | 4 | HMEJ | MII | male | yes | no | - | - | - |
| 20 | 5 | HMEJ | MII | female | yes | yes | mosaic | BC38F | BC70R |
| 20 | 6 | HMEJ | MII | female | yes | no | - | - | - |
| 20 | 7 | HMEJ | MII | male | yes | yes | hemi | BC38F | BC9R |
| 20 | 8 | HMEJ | MII | female | yes | no | - | - | - |
| 20 | 9 | HMEJ | MII | male | yes | no | - | - | - |
| 20 | 10 | HMEJ | MII | female | yes | yes | mosaic | BC40F | BC1R |
| 20 | 11 | HMEJ | MII | male | no | no | - | - | - |
| 20 | 12 | HMEJ | MII | female | yes | no | - | - | - |
| 20 | 13 | HMEJ | MII | female | yes | no | - | - | - |
| 20 | 14 | HMEJ | MII | female | no | no | - | - | - |
| 20 | 15 | HMEJ | MII | male | yes | yes | mosaic | BC40F | BC17R |
| 20 | 16 | HMEJ | MII | female | yes | yes | mosaic | BC40F | BC29R |
| 20 | 17 | HMEJ | MII | female | yes | no | - | - | - |
| 21 | 1 | HMEJ | MII | female | yes | yes | homo | BC40F | BC34R |
| 21 | 2 | HMEJ | MII | male | yes | yes | HII | BC40F | BC38R |
| 21 | 3 | HMEJ | MII | male | no | no | - | - | - |
| 21 | 4 | HMEJ | MII | male | yes | no | - | - | - |
| 21 | 5 | HMEJ | MII | female | yes | yes | homo | BC40F | BC40R |
| 21 | 6 | HMEJ | MII | male | yes | yes | mosaic | BC40F | BC48R |
| 21 | 7 | HMEJ | MII | female | no | no | - | - | - |
| 21 | 8 | HMEJ | MII | female | yes | no | - | - | - |
| 21 | 9 | HMEJ | MII | male | yes | no | - | - | - |
| 21 | 10 | HMEJ | MII | female | no | no | - | - | - |
| 21 | 11 | HMEJ | MII | male | yes | no | - | - | - |
| 21 | 12 | HMEJ | MII | male | yes | no | - | - | - |
| 22 | 1 | HMEJ | MII | female | no | no | - | - | - |
| 22 | 2 | HMEJ | MII | male | yes | no | - | - | - |
| 22 | 3 | HMEJ | MII | male | no | no | - | - | - |
| 22 | 4 | HMEJ | MII | female | yes | no | - | - | - |
| 22 | 5 | HMEJ | MII | male | yes | yes | mosaic | BC40F | BC52R |
| 22 | 6 | HMEJ | MII | fail | - | - | - | - | - |
| 22 | 7 | HMEJ | MII | male | no | no | - | - | - |
| 22 | 8 | HMEJ | MII | male | yes | no | - | - | - |
| 22 | 9 | HMEJ | MII | male | yes | no | - | - | - |
| 22 | 9 | HMEJ | MII | male | yes | no | - | - | - |
| 23 | 1 | HMEJ | MII | male | yes | no | - | - | - |
| 23 | 2 | HMEJ | MII | male | yes | no | - | - | - |
| 23 | 3 | HMEJ | MII | male | no | no | - | - | - |
| 23 | 4 | HMEJ | MII | fail | - | - | - | - | - |
| 23 | 5 | HMEJ | MII | male | no | no | - | - | - |
| 23 | 6 | HMEJ | MII | female | yes | no | - | - | - |
| 23 | 7 | HMEJ | MII | male | yes | no | - | - | - |
| 23 | 8 | HMEJ | MII | male | yes | yes | mosaic | BC40F | BC54R |
| 23 | 9 | HMEJ | MII | male | yes | no | - | - | - |
| 23 | 10 | HMEJ | MII | male | yes | no | - | - | - |
| 23 | 11 | HMEJ | MII | male | yes | no | - | - | - |
| 23 | 12 | HMEJ | MII | female | yes | no | - | - | - |
| 23 | 13 | HMEJ | MII | male | yes | yes | hemi | BC40F | BC62R |
| 23 | 14 | HMEJ | MII | male | yes | no | - | - | - |
| 23 | 15 | HMEJ | MII | female | no | no | - | - | - |
| 23 | 16 | HMEJ | MII | male | yes | yes | mosaic | BC40F | BC70R |
| 23 | 17 | HMEJ | MII | male | yes | no | - | - | - |
| 23 | 18 | HMEJ | MII | female | yes | yes | mosaic | BC40F | BC9R |
| 23 | 19 | HMEJ | MII | male | no | no | - | - | - |
| 23 | 20 | HMEJ | MII | fail | - | - | - | - | - |
| 23 | 21 | HMEJ | MII | male | no | no | - | - | - |
| 24 | 1 | HMEJ | 6hpi | male | yes | yes | hemi | BC48F | BC1R |
| 24 | 2 | HMEJ | 6hpi | male | yes | no | - | - | - |
| 24 | 3 | HMEJ | 6hpi | male | yes | no | - | - | - |
| 24 | 4 | HMEJ | 6hpi | male | yes | no | - | - | - |
| 24 | 5 | HMEJ | 6hpi | female | yes | no | - | - | - |
| 24 | 6 | HMEJ | 6hpi | female | yes | no | - | - | - |
| 24 | 7 | HMEJ | 6hpi | female | yes | yes | mosaic | BC48F | BC17R |
| 24 | 8 | HMEJ | 6hpi | male | yes | no | - | - | - |
| 24 | 9 | HMEJ | 6hpi | female | yes | yes | mosaic | BC48F | BC29R |
| 24 | 10 | HMEJ | 6hpi | female | yes | no | - | - | - |
| 24 | 11 | HMEJ | 6hpi | male | yes | no | - | - | - |
| 24 | 12 | HMEJ | 6hpi | female | yes | no | - | - | - |
| 24 | 13 | HMEJ | 6hpi | female | no | no | - | - | - |
| 24 | 14 | HMEJ | 6hpi | male | yes | no | - | - | - |
| 24 | 15 | HMEJ | 6hpi | male | yes | no | - | - | - |
| 24 | 16 | HMEJ | 6hpi | fail | - | - | - | - | - |
| 24 | 17 | HMEJ | 6hpi | male | yes | yes | HII | BC48F | BC34R |
| 24 | 18 | HMEJ | 6hpi | female | yes | yes | HII | BC48F | BC38R |
| 25 | 1 | HR | MII | male | no | no | - | - | - |
| 25 | 2 | HR | MII | male | yes | no | - | - | - |
| 25 | 3 | HR | MII | female | yes | no | - | - | - |
| 25 | 4 | HR | MII | male | no | no | - | - | - |
| 25 | 5 | HR | MII | male | yes | no | - | - | - |
| 25 | 6 | HR | MII | female | yes | no | - | - | - |
| 25 | 7 | HR | MII | fail | - | - | - | - | - |
| 25 | 8 | HR | MII | female | no | no | - | - | - |
| 26 | 1 | HR | MII | female | yes | no | - | - | - |
| 26 | 2 | HR | MII | male | yes | no | - | - | - |
| 26 | 3 | HR | MII | female | yes | yes | mosaic | BC48F | BC40R |
| 26 | 4 | HR | MII | female | no | no | - | - | - |
| 26 | 5 | HR | MII | female | yes | no | - | - | - |
| 26 | 6 | HR | MII | male | yes | no | - | - | - |
| 26 | 7 | HR | MII | female | no | no | - | - | - |
| 26 | 8 | HR | MII | male | yes | no | - | - | - |
| 27 | 1 | HR | MII | female | yes | no | - | - | - |
| 27 | 2 | HR | MII | male | no | no | - | - | - |
| 27 | 3 | HR | MII | male | yes | no | - | - | - |
| 27 | 4 | HR | MII | male | yes | yes | mosaic | BC48F | BC48R |
| 27 | 5 | HR | MII | male | yes | no | - | - | - |
| 27 | 6 | HR | MII | male | yes | no | - | - | - |
| 27 | 7 | HR | MII | male | yes | no | - | - | - |
| 27 | 8 | HR | MII | female | yes | yes | homo | BC48F | BC52R |
| 28 | 1 | HR | MII | female | yes | no | - | - | - |
| 28 | 2 | HR | MII | female | yes | no | - | - | - |
| 28 | 3 | HR | MII | male | yes | no | - | - | - |
| 28 | 4 | HR | MII | female | yes | yes | homo | BC48F | BC54R |
| 28 | 5 | HR | MII | male | yes | no | - | - | - |
| 28 | 6 | HR | MII | female | yes | yes | mosaic | BC48F | BC62R |
| 28 | 7 | HR | MII | male | yes | no | - | - | - |
| 29 | 1 | HR | 6hpi | female | yes | yes | mosaic | BC48F | BC70R |
| 29 | 2 | HR | 6hpi | female | yes | yes | mosaic | BC48F | BC9R |
| 29 | 3 | HR | 6hpi | male | yes | no | - | - | - |
| 29 | 4 | HR | 6hpi | female | yes | yes | mosaic | BC52F | BC1R |
| 29 | 5 | HR | 6hpi | female | yes | no | - | - | - |
| 29 | 6 | HR | 6hpi | female | yes | no | - | - | - |
| 29 | 7 | HR | 6hpi | male | yes | no | - | - | - |
| 30 | 1 | HMEJ | 6hpi | female | yes | yes | homo | BC52F | BC17R |
| 30 | 2 | HMEJ | 6hpi | male | yes | no | - | - | - |
| 30 | 3 | HMEJ | 6hpi | female | yes | yes | mosaic | BC52F | BC29R |
| 30 | 4 | HMEJ | 6hpi | male | yes | no | - | - | - |
| 31 | 1 | HMEJ | 6hpi | male | yes | yes | HII | BC52F | BC34R |
| 31 | 2 | HMEJ | 6hpi | male | yes | yes | mosaic | BC52F | BC38R |
| 31 | 3 | HMEJ | 6hpi | female | yes | no | - | - | - |
| 31 | 4 | HMEJ | 6hpi | male | yes | yes | HII | BC52F | BC40R |
| 31 | 5 | HMEJ | 6hpi | female | yes | yes | mosaic | BC52F | BC48R |
| 31 | 6 | HMEJ | 6hpi | male | yes | no | - | - | - |
| 31 | 7 | HMEJ | 6hpi | male | yes | yes | mosaic | BC52F | BC52R |
| 31 | 8 | HMEJ | 6hpi | male | no | no | - | - | - |
| 31 | 9 | HMEJ | 6hpi | female | yes | no | - | - | - |
| 32 | 1 | HR | 6hpi | male | yes | no | - | - | - |
| 32 | 2 | HR | 6hpi | female | yes | no | - | - | - |
| 32 | 3 | HR | 6hpi | male | yes | no | - | - | - |
| 32 | 4 | HR | 6hpi | male | no | no | - | - | - |
| 32 | 5 | HR | 6hpi | male | yes | no | - | - | - |
| 32 | 6 | HR | 6hpi | male | yes | no | - | - | - |
| 32 | 7 | HR | 6hpi | male | yes | no | - | - | - |
| 33 | 1 | HR | 6hpi | female | yes | no | - | - | - |
| 33 | 2 | HR | 6hpi | fail | - | - | - | - | - |
| 33 | 3 | HR | 6hpi | female | no | no | - | - | - |
| 33 | 4 | HR | 6hpi | fail | - | - | - | - | - |
| 33 | 5 | HR | 6hpi | fail | - | - | - | - | - |
| 33 | 6 | HR | 6hpi | female | yes | no | - | - | - |
| 33 | 7 | HR | 6hpi | female | yes | no | - | - | - |
| 33 | 8 | HR | 6hpi | female | yes | no | - | - | - |
| 34 | 1 | HR | 6hpi | female | yes | no | - | - | - |
| 34 | 2 | HR | 6hpi | male | yes | no | - | - | - |
| 34 | 3 | HR | 6hpi | female | yes | no | - | - | - |
| 34 | 4 | HR | 6hpi | female | yes | no | - | - | - |
| 34 | 5 | HR | 6hpi | male | yes | no | - | - | - |
| 34 | 6 | HR | 6hpi | male | no | no | - | - | - |
| 35 | 1 | HR | 6hpi | male | yes | no | - | - | - |
| 35 | 2 | HR | 6hpi | female | no | no | - | - | - |
| 35 | 3 | HR | 6hpi | female | yes | no | - | - | - |

**
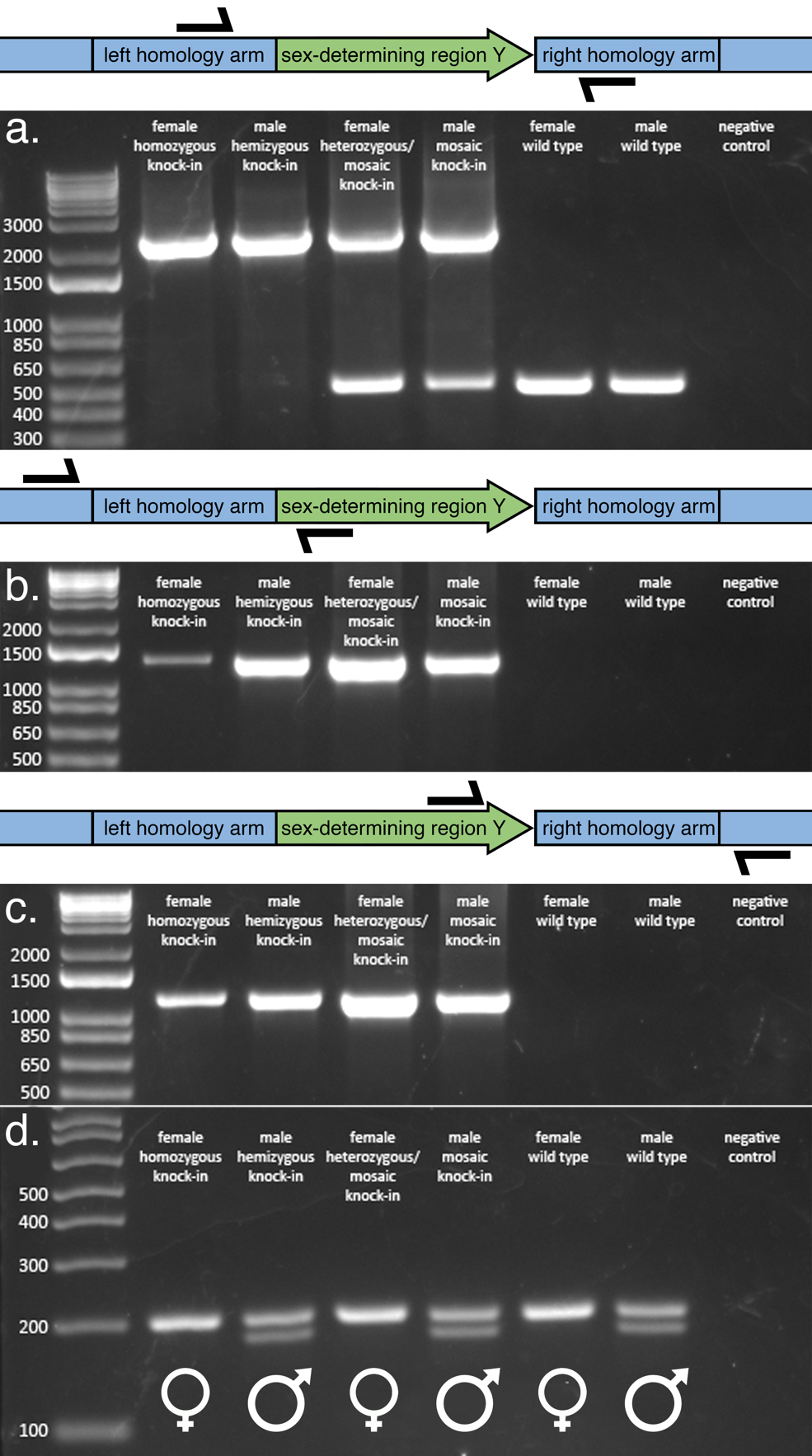
**

**Supplementary Figure S1.** PCR analysis of embryos for *SRY* knock-in at the ZFX locus. (a) Expected wild type product 520bp, knock-in product 2349bp. (b) Expected *SRY* knock-in product 1427bp. (c) Expected *SRY* knock-in product 1307bp. (d): female expected product 208bp, male expected product 189 and 208bp.

**
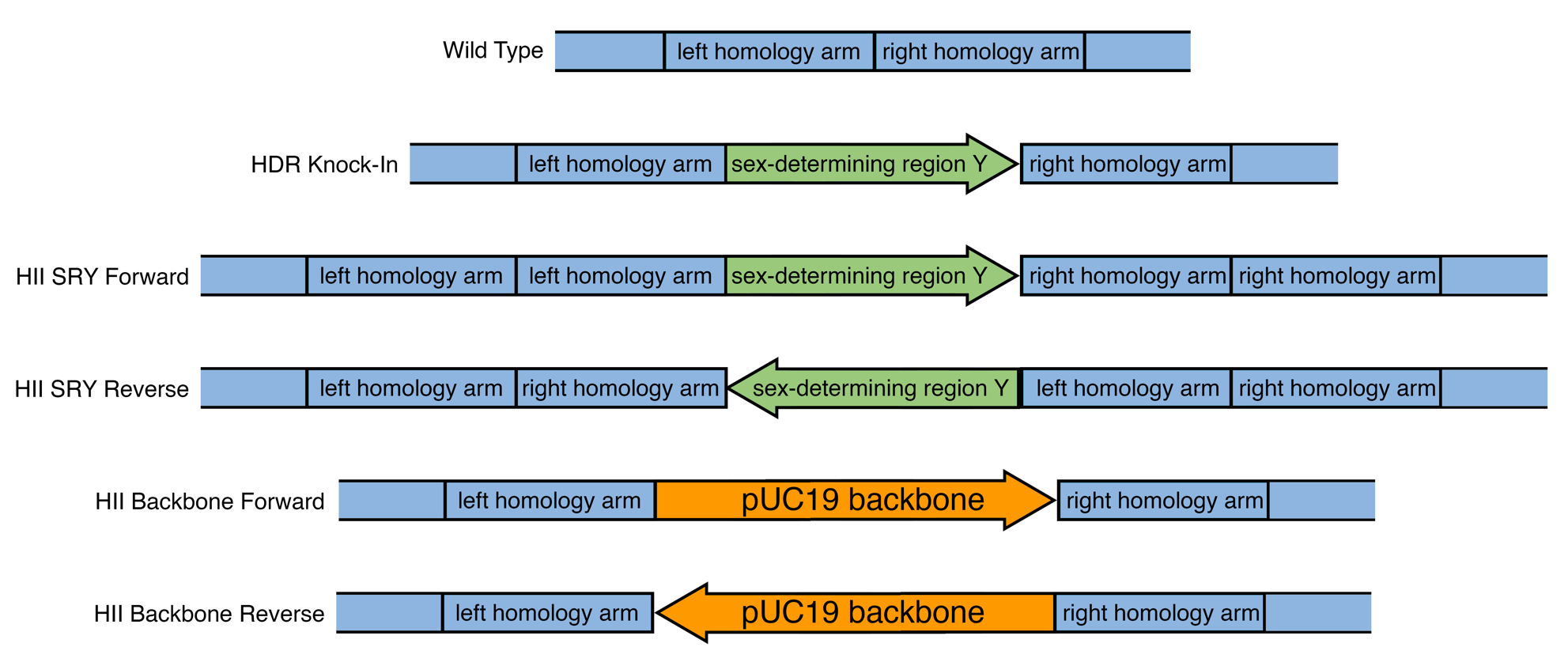
**

**Supplementary Figure S2.** Schematic representation of target sequences used to map PacBio reads against for evaluating presence of wildtype and knock-in of the sex-determining region Y (*SRY*) or plasmid backbone by homology directed repair (HDR) or homology independent repair (HII).
